# Interleukin-27-polarized HIV-resistant M2 macrophages are a novel subtype of macrophages that express distinct antiviral gene profiles in individual cells: implication for the antiviral effect via different mechanisms in the individual cell-dependent manner

**DOI:** 10.3389/fimmu.2025.1550699

**Published:** 2025-03-10

**Authors:** Tomozumi Imamichi, Jun Yang, Qian Chen, Suranjana Goswami, Mayra Marquez, Udeshika Kariyawasam, Homa Nath Sharma, Rosana Wiscovitch-Russo, Xuan Li, Akihiro Aioi, Joseph W. Adelsberger, Weizhong Chang, Jeanette Higgins, Hongyan Sui

**Affiliations:** ^1^ Laboratory of Human Retrovirology and Immunoinformatics, Frederick National Laboratory for Cancer Research, Frederick, MD, United States; ^2^ Laboratory of Basic Research, Septem-Soken, Osaka, Japan; ^3^ AIDS Monitoring Laboratory, Frederick National Laboratory for Cancer Research, Frederick, MD, United States

**Keywords:** interleukin-27, M2 macrophages, polarization, anti-viral genes, scRNA seq

## Abstract

**Introduction:**

Interleukin (IL)-27 is an anti-viral cytokine. IL-27-treated monocyte-derived macrophages (27-Mac) suppressed HIV replication. Macrophages are generally divided into two subtypes, M1 and M2 macrophages. M2 macrophages can be polarized into M2a, M2b, M2c, and M2d by various stimuli. IL-6 and adenosine induce M2d macrophages. Since IL-27 is a member of the IL-6 family of cytokines, 27-Mac was considered M2d macrophages. In the current study, we compared biological function and gene expression profiles between 27-Mac and M2d subtypes.

**Methods:**

Monocytes derived from health donors were differentiated to M2 using macrophage colony-stimulating factor. Then, the resulting M2 was polarized into different subtypes using IL-27, IL-6, or BAY60-658 (an adenosine analog). HIV replication was monitored using a p24 antigen capture assay, and the production of reactive oxygen species (ROS) was determined using a Hydrogen Peroxide Assay. Phagocytosis assay was run using GFP-labeled opsonized E. coli. Cytokine production was detected by the IsoPlexis system, and the gene expression profiles were analyzed using single-cell RNA sequencing (scRNA-seq).

**Results and Discussion:**

27-Mac and BAY60-658-polarized M2d (BAY-M2d) resisted HIV infection, but IL-6-polarized M2d (6-M2d) lacked the anti-viral effect. Although phagocytosis activity was comparable among the three macrophages, only 27-Mac, but neither 6-M2d nor BAY-M2d, enhanced the generation of ROS. The cytokine-producing profile of 27-Mac did not resemble that of the two subtypes. The scRNA-seq revealed that 27-Mac exhibited a different clustering pattern compared to other M2ds, and each 27-Mac expressed a distinct combination of anti-viral genes. Furthermore, 27-Mac did not express the biomarkers of M2a, M2b, and M2c. However, it significantly expressed CD38 (p<0.01) and secreted CXCL9 (p<0.001), which are biomarkers of M1.

**Conclusions:**

These data suggest that 27-Mac may be classified as either an M1-like subtype or a novel subset of M2, which resists HIV infection mediated by a different mechanism in individual cells using different anti-viral gene products. Our results provide a new insight into the function of IL-27 and macrophages.

## 1 Introduction

Interleukin (IL)-27 is a heterodimeric cytokine comprising the p28 and EBI3 subunits. p28 is structurally related to p35 of the alpha subunit of IL-12. Therefore, IL-27 is considered a member of the IL-12 cytokine family (1), along with IL-12, IL-23, IL-35, and IL-39 (2, 3). IL-27 binds to the IL-27 receptor (IL27R), which is composed of WSX1 and glycoprotein 130 (gp130) (4). Given that gp130 serves as a signal transducer of the IL-6 cytokine family, IL-27 is classified as an IL-6-family cytokine based on its receptor usage (4). IL-27 is primarily produced by antigen-presenting cells in response to stimulation by pattern recognition receptors. IL-27 promotes the proliferation of naïve CD4 T cells and differentiation of T helper 1 cells. IL-27 exerts regulatory effects on a multitude of T-cell subsets, including regulatory T cells and follicular helper T cells (5, 6). Multiple functions of this cytokine have been identified, such as promoting or curbing inflammatory diseases, cancers, aging, and viral infections (3, 6–11). It functions in multiple cell types (T cells, monocytes, macrophages, dendritic cells, neutrophils, hepatocytes, and keratinocytes) through IL27R (7, 12–14). Recently, IL-27 has been considered a biomarker for a certain infection of cancer (15–17). The binding of IL-27 to IL27R induces the Janus kinase/signal transducer and activator of transcription (JAK/STAT) signaling pathway. It functions as a pre-inflammatory, proinflammatory, or antiviral cytokine (7). The antiviral effect of IL-27 was first identified as a suppressor of human immune deficiency virus type 1 (HIV-1) infection in T cells and macrophages in culture supernatants of cervical cancer vaccine-treated peripheral blood mononuclear cells (PBMCs) (18), revealing the anti-HIV effect in dendritic cells (19). Additionally, it acts against different types of viruses such as influenza virus, hepatitis C virus, hepatitis B virus, cytomegalovirus coxsackievirus B3, respiratory syncytial virus, dengue virus, chikungunya virus, Zika virus, and Mayaro virus (13, 20–29).

IL-27 functions as a differentiation-inducing factor; it differentiates monocytes into HIV-resistant macrophages (30, 31) by enhancing the production of reactive oxygen species (ROS) (30, 32), inducing anti-HIV microRNAs (33–35) and autophagy (31).

Macrophages serve as phagocytes, produce ROS, show chemotaxis, secrete cytokines, present antigens for acquired immune responses, and play an important role in innate immunity (36–43). They are broadly divided into two groups: M1 macrophages and M2 macrophages (44–46). M1 macrophages (classical macrophages) are pro-inflammatory cells, and M2 macrophages are anti-inflammatory cells. M1 macrophages are activated from M0 cells by Toll-like receptor (TLR) ligands [pathogen-associated molecular patterns (PAMPs), such as lipopolysaccharide, or damage-associated molecular patterns (DAMPs), for example, High mobility group box-1 (HMGB1)], and S100 proteins or cytokines (such as interferon-γ) and then produce pro-inflammatory cytokines [such as tumor necrosis factor (TNF)-α, IL-1-α, IL-1β, IL-6, IL12, C-X-C motif chemokine ligand 9 (CXCL9), and CXCL10] (47), phagocytose, and initiate an immune response. M1 macrophages produce nitric oxide (NO) or ROS. NF-κB, STAT1, STAT5, IRF3, and IRF5 have been shown to regulate M1 cells and, as markers of M1, expression of TLR2, TLR4, CD80, CD86, and MHC-II (47). M2 macrophages are alternatively activated macrophage by exposure to certain cytokines such as IL-4, IL-10, IL-13, IL-33, and transforming growth factor beta (TGF-β) (48, 49), and regulated by STAT3, STAT6, and KLF4 (47–50). As M2 markers, CD163, CD206, CD209, FIZ1, and Ym1/2 are known (47, 50). M2 macrophages are subgrouped into M2a, M2b, M2c, and M2d (51). Recently, several novel macrophage subsets have been identified, including regulatory macrophages (Mreg), macrophage heme-related (Mhem), oxidized macrophages (Mox), and M3 and M4 macrophages (51–61).

Primary monocytes are differentiated into macrophages [monocyte-derived macrophages (MDMs)] using cytokines or serum. M1 and M2 macrophages are induced using granulocyte-macrophage colony-stimulating factor (GM-CSF) and macrophage colony-stimulating factor (M-CSF), respectively (44, 53, 62). M2 macrophages are further subdivided into M2a, M2b, M2c, and M2d based on polarization using different stimuli (47, 52); IL-4 and IL-13 have been shown to polarize M2 macrophages into M2a. Immunoglobulin complexes or Toll-like receptor ligands have been demonstrated to induce polarization of M2 into M2b. Moreover, IL-10, TGF-β, or glucocorticoid stimulation has been illustrated to induce M2c from M2 macrophages, and IL-6 stimulation has been demonstrated to polarize M2 macrophages into M2d (52, 63–65). Given that IL-27 shares gp130 to induce intracellular signaling as a member of the IL-6 family of cytokines, it was hypothesized that IL-27 treatment of M2 macrophages may be polarized into M2d-like cells. In our previous study, we examined the effects of IL-27 on the function of M2 macrophages. Our research demonstrated that IL-27-treated/polarized M2 macrophage (27-Mac) exhibited resistance to HIV replication and enhanced ROS production (32). While the enhanced ROS induction was associated with an increase in p47^phox^ expression (32), the mechanisms by which IL-27 suppressed HIV replication in 27-Mac remain unclear.

M2d is also induced by adenosine treatment (65–67). Adenosine stimulation induces intracellular signaling via adenosine receptors (ARs). ARs are members of the seven transmembrane receptors (68) and comprise four subclasses: A_1_, A_2A_, A_2B_, and A_3_ (69). A_1_ and A_3_ receptors couple to Gi/o proteins and inhibit the activity of adenylyl cyclase, whereas A_2A_ and A_2B_ preferentially couple to Gs proteins and stimulate cAMP formation (69–71). Although each receptor agonist has been developed and AR-mediated intracellular signaling and cellular function have been investigated, the function of adenosine-induced M2d on HIV replication is poorly understood. In the current study, to elucidate the function of 27-Mac and the mechanism of HIV resistance, comparative studies were performed using 27-Mac and IL-6-polarized M2d along with adenosine-polarized M2d. A multiplexed proteomic workflow and single-cell RNA sequencing (scRNA-Seq) were utilized in the study. Those systemic analyses further help to understand the distribution of each known antiviral gene expression in each cell. In summary, the current study provided new insights into the function of 27-Mac as a potent novel subset of macrophages resisting broad virus replication in individual cells with a different manner. Our results further support that IL-27 may serve as a therapeutic agent in cytokine-based therapy for the treatment of HIV and other viral infections.

## 2 Materials and methods

### 2.1 Cells and reagents

PBMCs were isolated from healthy donors’ apheresis packs (NIH blood bank) using a lymphocyte separation medium (ICN Biomedical, Aurora, OH, USA) (18); CD14(+) monocytes were separated from PBMCs using CD14 MicroBeads (Miltenyi Biotec, Auburn, CA, USA), according to the manufacturer’s instructions (30). The purity of the cell types was at least 90%, based on the flow cytometric analysis. Cell viability was determined using the trypan blue (Thermo Fisher Scientific, Waltham, MA, USA) exclusion method. The CD14(+) monocytes were cultured at 37°C and 5% CO_2_ with saturating humidity for 7 days to differentiate into macrophages (M2 macrophages) in the presence of M-CSF (R&D Systems, Minneapolis, MN, USA) in macrophage-serum free medium (M-SFM, Thermo Fisher Scientific) containing 50 μg/mL gentamicin (Thermo Fisher) and 10 mM 4-(2-hydroxyethyl)-1-piperazine ethane sulfonic acid (HEPES), pH 7.4 (Quality Biological, Gaithersburg, MD, USA) (72). After differentiation, M2 macrophages were maintained in the complete D-MEM medium (Thermo Fisher) supplemented with 10% (*v*/*v*) fetal bovine serum (FBS; R&D Systems), 10 mM HEPES, and 50 μg/mL gentamicin (D10 medium) as previously described (73). HEK293T cells were obtained from ATCC (Manassas, VA, USA) and maintained in the complete D-MEM (74). Recombinant human IL-6, IL-27, and interferon (IFN)-γ were purchased from R&D Systems. A non-selective AR agonist [5′-*N*-ethylcarboxamidoadenosine (NECA)], an A_1_AR specific agonist [2-chloro-*N* ^6^-cyclopentyladenosine (CCPA)], an A_2a_AR specific agonist (CGS21680), A_2b_AR specific agonist (BAY60-6583), and an A_3_AR specific agonist (piclidenoson: IB-MECA) were obtained from Cayman (Ann Arbor, MI, USA). Plasmid encoding, CCR5 tropic HIV-1 strain, HIV-1_AD8_ (75), was obtained from Dr. M. Martin (NIAID, Bethesda, MD, USA), and pNL4-3.Luc.R2.E2 (76, 77) was obtained from the NIH AIDS reagent program (ATCC). Plasmid was isolated using the EndoFree Plasmid Maxi isolation kit (Qiagen, Germantown, MD, USA).

### 2.2 Polarization of M2 macrophages

M2 macrophages were seeded onto culture plates at 0.15 × 10^6^ cells/cm^2^ cell seeding density in D10 medium, cultured at 37°C and 5% CO_2_ with saturating humidity overnight, and then polarized in the presence of 100 ng/mL IL-27, 30 ng/mL IL-6, or 10 μM BAY60-6583 for 3 days. After incubation, the polarized cells were washed with fresh D10 and used as subjects for assays. Cell viability was assessed using the trypan blue exclusion method using a Trypan Blue solution.

### 2.3 Preparation of viral stocks

The infectious HIV-1_AD8_ was prepared by transfection of pAD8 (75) to HEK293T cells (ATCC) using the TransIT-293 transfection kit (Mirus, Madison, WI, USA) as previously described (18, 30). Briefly, 6 × 10^6^ HEK293T cells were incubated in a 100-mm tissue culture dish in 10 mL D10 medium for 16 hrs, and then DNA-lipid complex (10 µg DNA and 30 µL of TransIT-293) in 600 µL of OPT-MEM (Thermo Fisher) was added dropwise. The cells were further incubated for 48 hrs; then, virus particles were isolated from the transfection supernatants using ultracentrifugation with 20% sucrose cushion at 100,000 × *g* for 2 hrs at 4°C (78), resuspended in 1/10 volume of the transfection supernatants in D10 medium, and stored as viral stocks at −80°C until use. The viral infection titer [50% tissue culture infectious dose (TCID50)] of the viral stock was determined by an endpoint assay by modifying a method described in our previous report (79). In the current study, TCID50 was quantified using M2 macrophage rather than T cells, and cells were cultured for 14 days (18). The infection titer was determined using the p24 antigen capture kit (PerkinElmer, Waltham, MA USA) with 50 pg/mL as a cutoff. A pseudotyped HIV, HIVLuc-VSV-G, was prepared by co-transfection of both pNL4.3.Luc.R2.E2 and pLTR-VSVG into HEK293T cells as previously described (14, 73), and virus amounts were quantitated using the p24 antigen capture kit.

### 2.4 HIV-1 infection and replication assay

Macrophages were seeded at 50 × 10^3^ cells/well in 96-well plates and cultured overnight at 37°C in D10 medium. The cells were then infected with 5000 TCID50 HIV-1_AD8_ [multiplicity of infection (MOI) = 0.01] for 2 hrs at 37°C and then washed three times with warm D10 medium (72, 73). The infected cells were cultured in 200 µL D10 medium for 14 days with half of the medium changed every 3 to 4 days with a fresh warm medium. All replication assays were conducted in quadruplicate, and HIV-1 replication was determined by measuring the p24 antigen in the culture supernatants using the p24 antigen capture assay kit (PerkinElmer, Boston, MA, USA).

### 2.5 Reactive oxygen species

The quantification of ROS was performed as previously described (32). Briefly, macrophages (50 × 10^3^ cells/well) were seeded in 96-well plates overnight at 37°C and then polarized using 30 ng/mL of IL-6, 100 ng/mL of IL-27, or 10 μM BAY for 3 days. The cells were washed with Krebs-Ringer Phosphate Buffer supplemented with 5.5 mM glucose (KRPB-G) and then stimulated with 100 ng/mL of phorbol myristate acetate (PMA) for 30 min at 37°C in Amplex™ Red Hydrogen Peroxide/Peroxidase Assay buffer. ROS induction was detected using the EnSpire Multimode Plate Reader (PerkinElmer).

### 2.6 Phagocytosis assay

Endogenous phagocytic activity was compared among M2, 27-Mac, 6-M2d, and BAY-M2d using the phagocytosis assay kit (Abcam, Waltham, MA, USA). Briefly, 50 × 10^3^ M2 macrophages were seeded in quadruplicate onto a 96-well plate and then cultured for 3 days at 37°C in the presence of D10 medium alone, 100 ng/mL of IL-27, or 30 ng/mL of IL-6 or 10 μM of BAY60-6583. The polarized cells were washed with warm D10 medium three times and then cultured with or without green fluorescent protein (GFP)-labeled opsonized *Escherichia coli* (T:E ratio at 10) for 3 hrs in D10. The cells were washed with phosphate-buffered saline (PBS) and then lysed using 2x radioimmunoprecipitation assay (RIPA) buffer (Boston BioProducts, Milford, MA, USA) supplemented with a cocktail of proteinase and phosphatase inhibitors (Pierce, Thermo Fisher Scientific) and EDTA (Pierce), and phagocytosed particles were quantified using EnSpire (PerkinElmer).

### 2.7 Western blotting

Western blotting (WB) was performed as previously described (78). Briefly, M2 macrophages (1.5 × 10^6^ cells/well in a 6-well plate) were cultured in D10 medium with or without 100 ng/mL IL-27, 30 ng/mL of IL-6, or IFN-gamma for 3 days. The cells were washed with cold PBS, lysed using 150 μL of 1x RIPA lysis buffer (Boston BioProducts) supplemented with 5 mM EDTA (Quality Biological, Gaithersburg, MD, USA) and 1x phosphate and protease inhibitor cocktail (Thermo Fisher Scientific), and incubated on ice for 15 min. Then, the cell debris-free cell lysates were obtained by centrifuging at 15,000 × *g* at 4°C for 10 min. Protein concentration was determined using the BCA protein assay kit (Pierce, Thermo Fisher Scientific), and 20 μg total protein for each lysate was subjected to 4%–12% NuPAGE Bis-Tris gels (Thermo Fisher Scientific). Proteins were transferred onto 0.2- or 0.45-μm pore-size nitrocellulose membranes (Thermo Fisher Scientific) and probed with antibodies against proteins of interest (antibodies are listed in **Supplementary Table S1**). Protein bands were detected using the ECL Prime Western Blotting Detection Reagent (Cytiva Life Sciences, Marlborough, MA, USA) with the Azure 300 (Azure Biosystems, Dublin, CA, USA) or Sapphire FL Biomolecular Imager (Azure Biosystems) (80). Band intensities were analyzed using Fiji (NIH ImageJ; http://rsbweb.nih.gov/ij/).

### 2.8 Quantitation of proteins in the culture supernatants

To compare the produced amounts of cytokines and C1q in the culture supernatants, M2 macrophages from five different donors were cultured at 37°C for 3 days in the presence of D10 medium alone, 30 ng/mL of IL-6, 100 ng/mL of IL-27, and 10 μM of BAY. The cells were washed and then cultured for 2 days in the absence of stimulus. To ascertain a potential correlation between the anti-HIV effect and the cytokine profiles, cells were cultivated in the same culture condition as HIV-infected cells. Subsequently, the cell-free culture supernatants were collected and stored at −20°C until use. Granzyme B, IFN-gamma, IL-10, IL-13, IL-17A, IL-2, IL-4, IL-5, IL-6, IL-7, IL-8, IL-9, IP-10, monocyte chemoattractant protein-1 (MCP1/CCL2), macrophage inflammatory protein (MIP)-1 alpha (MIP-1α/CCL3), MIP-1 beta (MIP-1β/CCL4), TNF-α, and TNF-β were quantified using the IsoLight automation system (Bruker Cellular Analysis, Emeryville, CA, USA). Briefly, the frozen supernatants were thawed at room temperature for 30 min and mixed well by pipetting up and down prior to loading in the system. An aliquot of 5.5 μL of each sample was pipetted into each microchamber of a CodePlex chip pre-patterned with a 22-plex antibody with the IsoPlexis Human Adaptive Immune Panel (Bruker Cellular Analysis). Each sample was run in duplicate. D10 medium was used as background control. The chips were sealed and then loaded into the IsoLight automation system, and various cytokine proteins were measured automatically by fluorescence ELISA and analyzed by the IsoSpeak software (Bruker Cellular Analysis). Quantification of TGF-β1 was conducted using TGF-β1 ELISA (R&D Systems) with the reactivation buffer (R&D Systems) following the vendor’s protocol. The resulting cytokine amounts were plotted using a heat map created using GraphPad Prism version 9.3.1 (Boston, MA, USA). The amounts of IFN-α, IFN-β, and C1q in the culture supernatants were quantified using all type IFN-α ELISA kits (PBL Assay Science, detection limit <6.25 pg/mL), Verikine-HS Human IFN beta ELISA kit (PBL Assay Science, detection limit <1.2 pg/mL), and Human C1q ELISA kit (Invitrogen, Thermo Fisher Scientific, detection limit 0.9 ng/mL), respectively. All assays were conducted following the manufacturer’s protocols.

### 2.9 Construction of scRNA-Seq libraries

M2 macrophages (4 × 10^6^ cells) were seeded in 60-mm Petri dishes and cultured at 37°C for more than 16 hrs in D10 media. The cells were polarized with IL-6, BAY60-6583, or IL-27 for 3 days; washed three times with the warmed PBS; and then incubated with 2.5 mL of trypsin-EDTA (0.25%) (QBI) at 37°C for 15 min in a CO_2_ (5%) incubator. After the incubation, 5 mL of warm D10 was added per dish, and the cells were detached by flushing the medium. Cells were washed three times with D10 media, and cell viability and cell number were determined using Cellometer Auto 2000 (Nexcelom Bioscience, Lawrence, MA, USA) with the ViaStain AOPI Staining Solution (Nexcelom Bioscience). Consistently, the cell viability was 98% to 100%. Each cell was resuspended at 1,000 cells/µL in D10. The scRNA-Seq libraries were constructed using the Chromium Next GEM Single Cell 5′ Reagent Kit V2 (Dual Index) (10x Genomics, Pleasanton, CA, USA) following the manufacturer’s instructions. Briefly, 16.5 µL of the cell suspension with the master mix containing reverse transcription (RT) reagent and template switch oligos and RT enzyme was loaded into a Chromium Chip K (10x Genomics). The single-cell GEM generation and barcoding followed by cDNA synthesis were run on the Chromium Chip K in the 10x Chromium Controller (10x Genomics). The cDNA was amplified by 13 cycles of reaction. Based on Agilent 2100 Bioanalyzer (Agilent Biotechnology, Santa Clara, CA, USA) and Qubit 4 analysis (Thermo Fisher Scientific), the scRNA-Seq libraries contained a single peak of DNA between 300 and 1,000 bp (average fragment sizes were 450–550 bp).

### 2.10 Analysis of scRNA-Seq

Sequence data were processed using Cell Ranger V6.0.1 (10x Genomics). The resulting count matrices followed the standard pipeline with default parameters. The count matrices for each sample were loaded into Partek Flow (version 12.3.1, https://www.partek.com) for further quantification and statistical analysis. All data underwent quality control (QC) and were filtered based on the following criteria. 1) Low-quality cells and potential doublets were filtered out from analysis using the following parameters: total reads per cell (800–40,000), expressed genes per cell (800–6,000), and mitochondrial reads (<15%). 2) The genes (features) with maximum values ≤ 1.0 were excluded. Counts were normalized by counts per million, added with 1.0, and then transformed to log 2.0. The top 20 of the principal component analysis (PCA) contributing to data variance were used for unbiased clustering (graph-based clustering) and presented using Uniform Manifold Approximation and Projection (UMAP), a dimensional reduction algorithm that shows groups of similar cells as clusters on a scatter plot. Differential gene expression analysis among the receptor-positive cells for each macrophage subtype was performed using an analysis of variance (ANOVA) model; a gene is considered differentially expressed if its *p* ≤ 0.05 and absolute fold change ≥3. The bubble plot was performed using the Partek Flow, and the gene annotation and function analysis for the selected gene lists were performed in DAVID (81) and Metascape (82).

### 2.11 Flow cytometric analysis

The cellular phenotype was confirmed by flow cytometric analysis as previously described (14). A total of 1 × 10^6^ cells of M2, 27-Mac, 6-M2d, and BAY-M2d macrophages were washed three times with ice-cold Dulbecco’s PBS (Thermo Fisher Scientific) in the presence of bovine serum albumin (BSA) and then blocked using Fc Receptor Blocker (Innovex Biosciences, Richmond, CA, USA) for 30 min at room temperature in the dark. Cells were washed twice in 2% BSA (MilliporeSigma, St. Louis, MO, USA) with 0.5% NaN3 (MilliporeSigma) in Dulbecco’s PBS (DPBS-BSA-NaN_3_). Cells were then stained, including paired isotype controls, for 15 min at room temperature in the dark. The following antibodies and their respective fluorochrome were used: CD38 (Fluorochrome PE-Cy7; Cat. # 560677, BD Biosciences, Franklin Lakes, NJ, USA), isotype control (Fluorochrome PE-Cy7; Cat. # 557872, BD Biosciences), CD130/GP130 (Fluorochrome AF647, Cat. # 564151, BD Biosciences), isotype control (Fluorochrome AF647, Cat. # 557714, BD Biosciences), WSX1 (Fluorochrome PE; Cat. # FAB14791PO2, R&D Systems), and isotype control (Fluorochrome PE; Cat. # IC0041P, R&D Systems). The cells were washed twice in DPBS-BSA-NaN_3_ and then run immediately on an LSRFortessa flow cytometer (BD Biosciences). The results were analyzed using FCS Express version 7 (*De Novo* Software, Pasadena, CA, USA) (14).

### 2.12 Statistical analysis

Intergroup comparisons were performed by one-way ANOVA with multiple comparison analysis or Student’s *t*-test using GraphPad Prism 9 (GraphPad, San Diego, CA, USA). *p*-Values less than 0.05 were considered statistically significant: * *p* < 0.05, ** *p* < 0.01, *** *p* < 0.001, **** *p* < 0.0001, and *p* > 0.05 was considered not significant (n.s.).

## 3 Results

### 3.1 Comparison of IL-27 and IL-6 treatment on HIV replication and STAT activation

IL-6 enhances HIV replication in a combination of M-CSF and GM-CSF-induced MDMs (83) and macrophage-like cell lines (84), whereas IL-27 suppresses HIV in M2 macrophages (32, 72).

**Figure 1:**
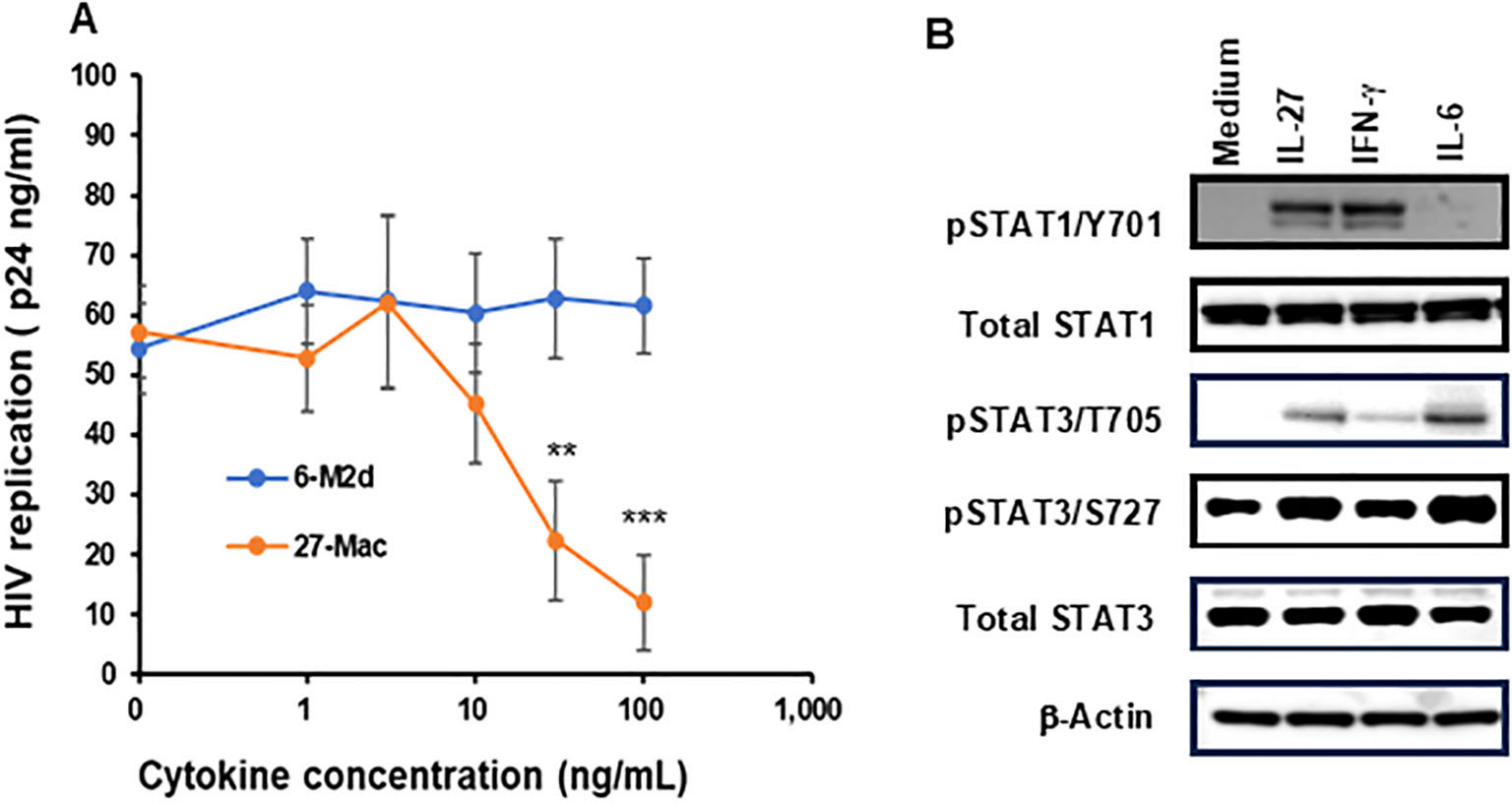
Comparison of HIV inhibitory effects of IL-27, IL-6, and adenosine receptor agonist-polarized M2 macrophages. **(A)** M2 macrophages were polarized in the presence of different concentrations of IL-6 or IL-27 for 3 days. The polarized cells were then infected with HIVAD8 as described in Materials and Methods and then cultured for 14 days. HIV replication was monitored using the p24 HIV antigen ELISA kit. **(B)** The STAT activation profile was compared. M2 macrophages were incubated with medium alone, 100 ng/mL IL-27, 100 ng/mL IFN-γ or 30 ng/mL IL-6 for 15 min, and then Western blotting was performed to measure the phosphorylation status of STAT-1 and STAT-3 in the cells. For each phosphorylated STAT measured, the unphosphorylated total protein was also measured. β-Actin was measured as a loading control. Data are mean ± SD (n = 4). IFN-γ **<0.01, ***<0.001.

To compare the HIV resistance between 27-Mac and 6-M2d, M2 macrophages were polarized with different amounts of IL-6 or IL-27 for 3 days, and the polarized cells were infected with replication-competent HIVAD8 (**Figure 1A**). HIV replication capacity was assessed by measuring HIV p24 levels in culture supernatants 14 days after infection using the p24 capture assay. HIV replication was dose dependently suppressed in 27-Mac. 27-Mac polarized with 30 ng/mL and 100 ng/mL of IL-27 suppressed HIV replication to 39.1% ± 0.7% (*p* < 0.01) and 21.0% ± 0.8% (*p* < 0.001), respectively, when compared to HIV replication in unpolarized cells. In contrast, 6-M2d did not indicate HIV resistance, suggesting that 27-Mac differs from 6-M2d in anti-HIV effect.

To define the difference between 27-Mac and 6-M2d at the initiation of cell activation, STAT activation was compared. M2 macrophages were stimulated for 15 min with each cytokine, and STAT activation was detected using WB. IL-27 induces the activation of STAT1 and STAT3 (13, 19, 20, 72, 85, 86); therefore, IFN-γ was used as a positive control for STAT1 activation. IL-27 and IL-6 induced phosphorylation of STAT3 at T705 and S727; however, IL-27 notably induced STAT1 phosphorylation at Y701, as did IFN-γ. IL-6 promoted the activation at a lower level of STAT1 activation than that by IL-27 or IFN-γ (**Figure 1B**). Amounts of each phosphorylated STAT were quantified using ImageJ, and one-way ANOVA statistical analysis was performed. The results showed that the phosphorylation at STAT1 Y701 by IL-27 was increased by 5.1 ± 1.2-fold compared to that by medium alone, and the activation level was compared to that by IFN-γ, but it was significantly higher than that by IL-6 (*p* < 0.01). On the contrary, the activation of STAT3 by IL-27 at T705 and S727 was increased by 141 ± 10.9- and 1.98 ± 0.09-fold, respectively, and their activation was significantly higher than that by IFN-γ (*p* < 0.01); however, it was lower than that by IL-6 (*p* < 0.01) (**Supplementary Figure S1**). These results indicated that IL-27 induced activation of STAT1 like IFN-γ did and modest activation of STAT3, suggesting that the downstream of the gene expression profile may differ between IL-27 and IL-6 treatment; consequently, 27-Mac differs from 6-M2d in the anti-HIV effect. Since IL-6 had no effect on HIV replication, STAT3 activation and genes induced by the activated STAT3 in the cells may not be involved in the anti-HIV effect.

### 3.2 A_2B_AR agonist and BAY60-6583-polarized M2 macrophages resisted HIV replication

**Figure 2:**
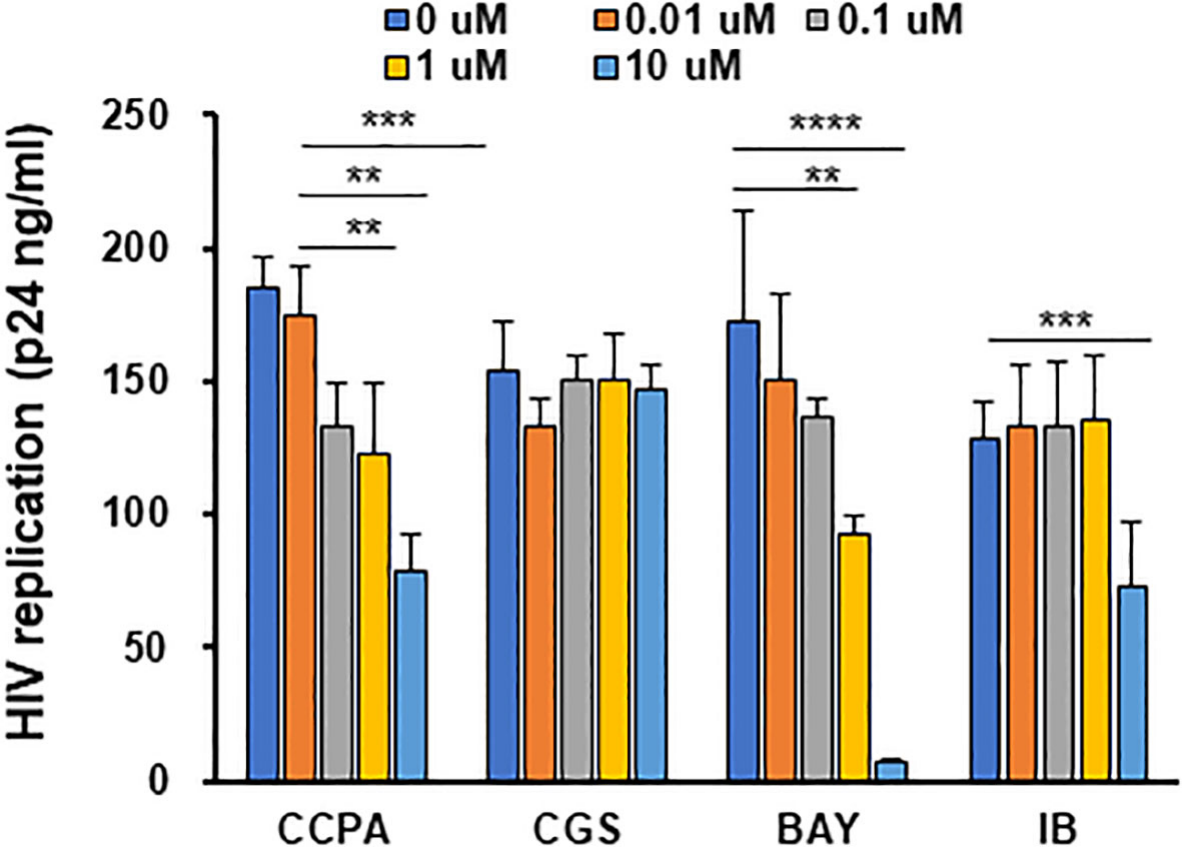
Adenosine analog-polarized macrophages suppress HIV infection. Evaluation of the anti-HIV effect among cells polarized with different adenosine receptor agonists. M2 macrophages were cultured for 3 days with different concentrations (0–10 μM) of adenosine receptor agonists. The agonists used for A_1_AR and A2_a_aAR were CGS 21680 (CCPA) and CGS 21680 (CGS), respectively; the agonist used for A2bR was BAY60-6583 (BAY); the agonist used for AR3 was IB-MECA/CF-101 (IB). The polarized cells were then infected with HIVAD8 and cultured for 14 days. HIV replication was monitored by p24 antigen ELISA. Results are representative of data from three independent experiments. (E and F) Evaluation of phagocytic activity and ROS-inducing activity of cells polarized with different stimuli. M2 macrophages were cultured for 3 days in the presence of medium alone, 100 ng/mL IL-27, 30 ng/mL IL-6, and 10 μM of BAY. ROS, reactive oxygen species. **: p<0.01, ***: p<0.001, ****:p<0.0001.

Adenosine also polarizes M2 macrophages into M2d (63, 65). Although adenosine is considered to have therapeutic potential, its impact on HIV replication in macrophages has been poorly investigated (87, 88). Adenosines bind to four different ARs—A_1_AR, A_2a_AR, A_2b_AR, and A_3_AR (69)—and induce the activation of adenylate cyclase (69, 89–92). Therefore, to define the role of each AR on viral replication, each AR specific agonist was used for polarization, and then HIV resistance was evaluated. M2 macrophages were cultured for 3 days with different doses (0–10 μM) of CCPA (90, 93) for A_1_AR, CGS 21680 (90) for A_2A_AR, BAY60-6583 for A_2b_AR, and IB-MECA/CF-101 (90, 94, 95) for A_3_AR, and the anti-HIV effect was monitored. BAY60-6583-polarized M2d (BAY-M2d) significantly suppressed HIV replication (*p* < 0.01 at 1 μM and *p* < 0.001 at 10 μM) without any impact on cell viability (**Figure 2**). Therefore, 10 μM BAY-M2d was chosen for further functional analysis using 27-Mac and 6-M2d.

### 3.3 Comparison of biological function among 27-Mac, 6-M2d, and BAY-M2d

**Figure 3:**
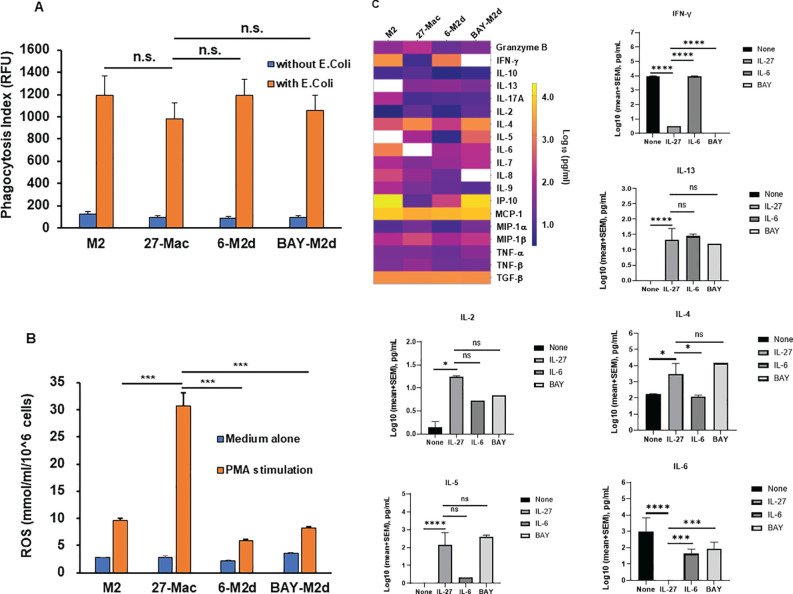
Comparison of phagocytic activity, ROS-inducing activity, and cytokine production among 27-Mac, 6-M2d, and BAY-M2d. M2 macrophages were cultured for 3 days in the presence of medium alone, 100 ng/mL IL-27, 30 ng/mL IL-6, and 10 μM of BAY. Then, **(A)** the polarized cells were cultured with or without GFP-labeled opsonized *Escherichia coli* (T:E ratio at 10) for 2 hrs at 37°C. The cells were washed, and the uptake of *E. coli* was determined using a fluorescence plate reader. Results are representative of three independent assays. Data are mean ± SD (n = 4). **(B)** Polarized cells were washed with D-PBS-glucose (PBS-G) and then stimulated with 100 nM PMA in PBS-G for 30 min at 37°C in the presence of Amplex Red Hydrogen Peroxide/Peroxidase Assay Reagent. Induced ROS amounts were determined as described in Materials and Methods. All assays were run in quadruplicate, and results are representative of data from three independent assays. **(C)** Macrophages from five independent donors were polarized for 3 days in the presence of D10 medium alone, 100 ng/mL IL-27, 30 ng/mL IL-6, or 10 μM of BAY, and then culture supernatants were collected. The cell-free culture supernatants were subjected to cytokine assay using the IsoPlexis Human Adaptive Immune Panel Kit for detecting IFN-γ IL-10, IL-13, IL-17A, IL-2, IL-4, IL-5, IL-6, IL-7, IL-8, IL-9, IP-10, MCP-1, MIP-α, MIP-1β, TNF-α, and TNF-β, and TGF-β ELISA Kit for TGF-β. The results (log pg/mL) were displayed by means of a heat map. White boxes indicate no cytokine detection. Bar graphs show each cytokine production with statistical analysis using one-way ANOVA. *p < 0.05, ***p<0.001, ****p<0.0001 and n.s., not significant. ROS, reactive oxygen species; GFP, green fluorescent protein; PBS, phosphate-buffered saline; PMA, phorbol myristate acetate.

To further characterize 27-Mac, biological function (phagocytosis, ROS induction, and cytokine production) was compared among three macrophages. Phagocytic activity was compared using opsonized fluorescence-tagged *E. coli* (30), and ROS-inducing activity was measured by PMA stimulation (32). Phagocytic activity was similar among the three cell types (**Figure 3A**); however, ROS induction was enhanced in only 27-Mac by sixfold (*p* < 0.01) compared to M2 macrophages (**Figure 3B**), indicating that 27-Mac differed from 6-M2d and BAY-M2d in ROS-inducing potential.

To elucidate the similarity of 27-Mac with macrophage subtypes, the profiles of cytokine secretion from four different macrophages (M2, 27-Mac, 6-M2d, and BAY-M2d) derived from five independent donors were analyzed. M2 macrophages or 27-Mac were cultured for 2 days in a D10 medium without any reagents, and the supernatants were collected. Cytokine levels in the culture supernatants were measured using an IsoLight automation system with the IsoPlexis Human Adaptive Immune Panel kit and a TGF-β ELISA kit. Cytokine production by each cell type was compared with that of unpolarized M2 macrophages. Unpolarized M2 macrophages produced granzyme B, IFN-γ, IL-10, IL-17A, IL-2, IL-4, IL-5, IL-6, IL-7, IL-8, IL-9, IP-10, MCP-1, MIP-1α, MIP-1β, TNF-α, TNF-β, and TGF-β (**Figure 3C**). Statistical analysis using one-way ANOVA demonstrated that 27-Mac significantly suppressed the production of IFN-γ (*p* < 0.0001) and IL-6 (*p* < 0.0001) and increased the secretion of IL-13 (*p* < 0.0001), IL-2 (*p* < 0.05), IL-4 (*p* < 0.05), and IL-5 (*p* < 0.0001), compared to unpolarized M2 macrophages. In the comparison of the cytokine profile between 27-Mac and 6-M2d, even though the heat map demonstrated a difference in the production of granzyme B, IFN-γ, IL-10, IL-2, IL-4, IL-5, IL-6, IL-7, IL-8, IP-10, MIP-α, and MIP-β, only the production of IFN-γ, IL-4, and IL-6 was significantly different. When the cytokine profile of 27-Mac was compared with that of 6-M2d, the production of IFN-γ, IL-4, and IL-6 was significantly different. On the contrary, when the profile was compared between 27-Mac and BAY-M2d, IFN-γ and IL-6 production was significantly distinct, but not for IL-4 production. Therefore, the profile of 27-Mac was similar to that of 6-M2d and BAY-M2d, but subtle differences exist in the profile. Of note, TGF-β and MCP-1 were produced regardless of polarization. Although TGF-β and MCP-1 have been reported as potential inhibitors of HIV infection (96, 97), they may not play a role in our study because both proteins were produced independently of polarization.

### 3.4 Gene expression profile of 27-Mac districts from others

**Figure 4:**
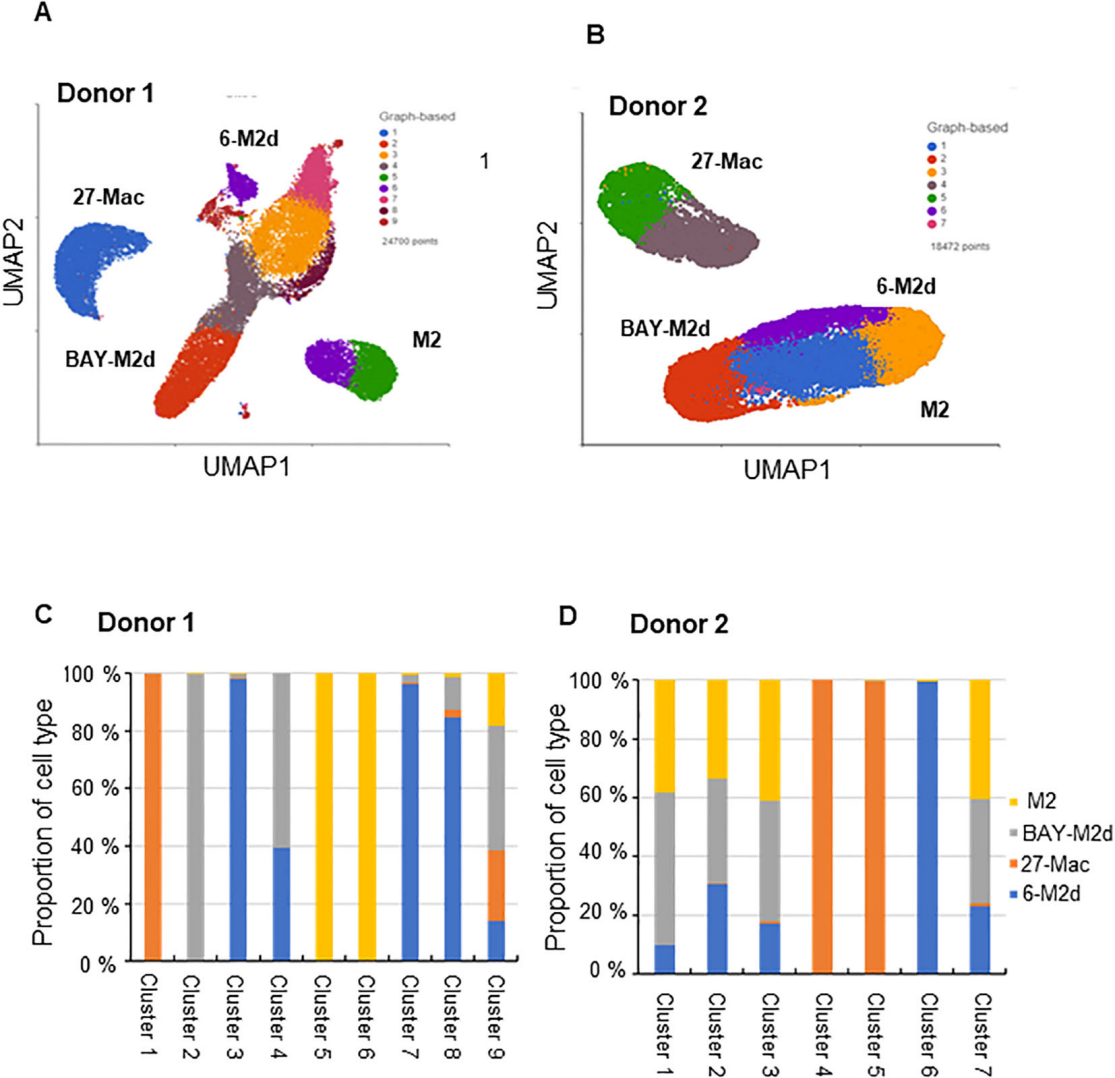
Single-cell RNA sequence analysis. The scRNA-Seq using fresh M2 macrophages, 27-Mac, 6-M2d, and BAY-M2d derived from two independent donors, Donor 1 **(A, C)** and Donor 2 **(B, D)**, was conducted as described in Materials and Methods. **(A, B)** UMAP was used for clustering and visualization; **(C, D)** the bar plots show the cell count in the graph-based cluster for each subtype. The cells with similar gene expression profiles were clustered closer together using PCA and graph-based clustering. Macrophages from Donor 1 and Donor 2 were distributed in a total of nine and six clusters, respectively. UMAP, Uniform Manifold Approximation and Projection; PCA, principal component analysis.

A comparative analysis of anti-HIV activity, induction of ROS generation, and cytokine production of 27-Mac with those of M2 macrophages, 6-M2d, and BAY-M2d revealed notable differences. To further characterize 27-Mac, scRNA-Seq was used to compare the gene expression profiles of the four different cell types. Polarized macrophages from two independent donors were analyzed. During QC, cells with low-quality scRNA-Seq data were excluded using the following thresholds: total reads per cell (800–40,000), expressed genes per cell (800–6,000), and mitochondrial reads (<15%). Additionally, genes with maximum values ≤1.0 were omitted. This process yielded 14,419 genes (features), 24,700 cells from Donor 1, and 18,472 cells from Donor 2. Of the 24,700 cells from Donor 1, 4,856 were identified as M2, 5,572 as 27-Mac, 7,878 as 6-M2d, and 6,394 as BAY-M2d. In total, 18,472 cells from Donor 2 were classified as follows: 4,205 as M2, 5,530 as 27-Mac, 3,905 as 6-M2d, and 4,832 as BAY-M2d (**Supplementary Table S2**). These cells were then subjected to further analyses. The UMAP method was employed to cluster and visualize gene distribution (**Figures 4A, B**). The UMAP results indicated that within the dataset derived from Donor 1, 27-Mac, 6-M2d, and BAY-M2d were clustered in each group and exhibited clear separation from M2 macrophages. In the case of Donor 2, 27-Mac, 6-M2d, and BAY-M2d were clustered in each group. However, some 6-M2d and BAY-M2d overlapped with M2 macrophages. Consequently, regarding gene expression profiles, the profile of 27-Mac was consistently distinguished from the other three cell types. In contrast, the profiles of 6-M2d and BAY-M2d indicated donor dependence, being either similar to or distinct from M2 macrophages.

The results of the gene expression analysis are presented as bar plots (**Figures 4C, D**). The graphs show cell populations in each cluster. PCA and graph-based clustering were used to cluster cells with similar gene expression profiles. A total of nine and seven distinct clusters were noticed in all cell types of Donor 1 and Donor 2, respectively. In Donor 1, M2 macrophages (yellow bars) constituted 96.8% of total cells and were separated into two clusters (clusters 5 and 6) (**Figure 4C**, **Supplementary Table S2**). In contrast, 27-Mac (orange bars) was mapped to a single cluster 1 (95.8% of 27-Mac). The majority of 6-M2d (98.7% of 6-M2d, indicated by blue bars) was distributed into four clusters (clusters 3, 4, 7, and 8), and 91.8% of BAY-M2d (shown in gray) was distributed in clusters 2 and 4. Both 6-M2d and BAY-M2d were present in cluster 4, whereas a small percentage of these two cell types (<0.01%) were distributed in cluster 1. Cluster 9, in contrast, was distributed across all four cell subtypes. In Donor 2 (**Figure 4D**), 98.9% of M2 macrophages (yellow bars) were distributed across three clusters (clusters 1, 2, and 3; **Supplementary Table S2**), whereas the majority (99.4%) of 27-Mac (orange bars) was distributed into two clusters (55.5% and 43.9% of 27-Mac were mapped in clusters 4 and 5, respectively). The gene expression profiles of 6-M2d (blue bars) and BAY-M2d (gray bars) were similar to those of M2 macrophages, which had been mapped into three clusters (clusters 1, 2, and 3). To identify functional differences in each cluster, biomarker genes (BGs) for each cluster were computed, and the functional enrichment analysis was performed using Metascape (82). In this analysis, BGs for each cluster were selected with the gene expressing more than threefold compared to those of other clusters, and then they were subjected to the biological function enrichment analysis. In the analysis, clusters composed of more than 85% of a single subtype were considered signature clusters for each subtype; in Donor 1, clusters 1 and 2 were considered signature clusters for 27-Mac and BAY-M2d, respectively (**Figure 4C**), and clusters 3, 7, and 8 were considered 6-M2d. The BGs in clusters 1 and 2 were involved in innate immunity and B-cell proliferation, respectively (**Supplementary Figure S2**). BGs of clusters for 6-M2d were involved in leukocyte chemotaxis, NABA matrisome associated, and chromosome segregation (**Supplementary Figures S3, S4**). In Donor 2, clusters 4 and 5 were considered the signatures of 27-Mac, and cluster 6 was the signature of 6-M2d (**Figure 4D**). BGs of clusters 4 and 5 were involved in chemotaxis and interferon signaling, respectively, and the BGs of cluster 6 were related to cytokine signaling in the immune system (**Supplementary Figures S5, S6**). Therefore, the functional enrichment analysis also demonstrated that 27-Mac differed from other M2d macrophages. These data indicated that the gene expression profile of 27-Mac differed from that of 6-M2d and BAY-M2d cell types, suggesting the potential expression of unique genes in 27-Mac.

### 3.5 Identification of 27-Mac gene markers

**Figure 5:**
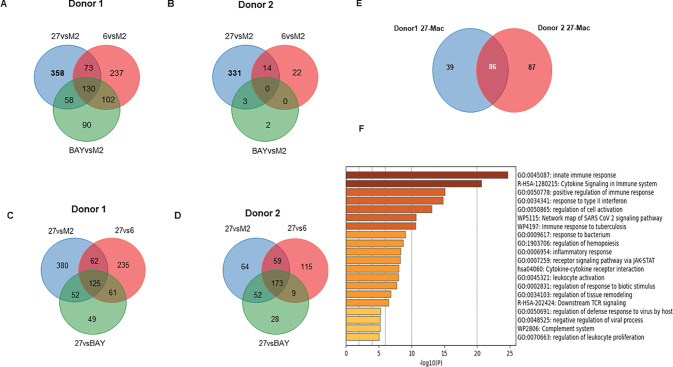
Comparison of the gene expression profiles among M2, 27-Mac, 6-M2d, and BAY-M2d. **(A, B)** To identify DEGs in each polarized cell of Donors 1 and Donor 2, gene expression profiles in each subset were compared to those in M2 macrophages using Venn diagram analysis. **(C, D)** To identify DEGs that are uniquely expressed in 27-Mac of Donor 1 **(C)** and Donor 2 **(D)** compared to polarized cells, a Venn diagram analysis was performed among DEGs of 27-Mac compared to f M2 (27vsM2), 27-Mac compared to 6-Mac (27vs6), and 27-Mac compared to BAY-M2d (27vsBAY). **(E)** A Venn diagram analysis to identify common DEGs in the gene uniquely expressed in 27-Mac of Donor 1 and Donor 2: 125 genes from Donor 1 and 172 genes from Donor 2. **(F)** Functional enrichment analysis by Metascape (Metascape.org) for 86 genes.

To identify the uniquely expressed genes in 27-Mac, the differentially expressed genes (DEGs) in 27-Mac, 6-M2d, and BAY-M2d were defined by comparing the normalized reads of each gene in each cell type with those of the corresponding genes in M2 macrophages (27-Mac vs. M2, 6-M2d vs. M2, and BAY-M2d vs. M2). The selection criteria were *p*-values <0.05 and absolute fold changes >3. In Donor 1, the numbers of DEGs in 27-Mac, 6-M2d, and BAY-M2d were 619, 542, and 380, respectively (column 1 of **Table 1** and **Supplementary Table S3**). In Donor 2, the numbers of DEGs in 27-Mac, 6-M2d, and BAY-M2d were 348, 36, and 5, respectively (column 5 of **Table 1** and **Supplementary Table S4**). A Venn diagram analysis revealed that 358 genes in the 27-Mac of Donor 1 (**Figure 5A**) and 331 genes in the 27-Mac of Donor 2 (**Figure 5B**) were unique DEGs in the 27-Mac, and 130 genes in the DEG of the 27-Mac were common DEGs among the three polarized cell types of Donor 1. It is noteworthy that no genes were common in all three types of Donor 2. To elucidate whether the genes that were uniquely expressed in 27-Mac could serve as gene markers, the gene profiles in 27-Mac were compared to the corresponding genes in 6-M2d or BAY-M2d. A total of 483 and 287 were identified as DEGs in 27-Mac of Donor 1 when compared to those in 6-M2d and BAY-M2d, respectively (column 2, **Table 1**, **Supplementary Table S5**). A total of 356 and 253 genes were identified as DEGs in 27-Mac of Donor 2 when compared to those in 6-M2d and BAY-M2d, respectively (column 6, **Table 1**, **Supplementary Table S6**). The gene lists were used together with DEGs in 27-Mac compared to those in M2 macrophages (619 in Donor 1 and 348 in Donor 2) in a Venn diagram analysis. Consequently, comparing 27-Mac to M2, 27-Mac to 6-M2d, and 27-Mac to BAY-M2d, 125 and 173 genes were identified as uniquely expressed genes in Donors 1 and 2, respectively (**Figures 5C, D**), To define commonly differentiated genes in 27-Mac of Donors 1 and 2, further analyses were performed using a Venn diagram. The results demonstrated that 86 genes (71 genes were upregulated, and 15 genes were downregulated) were common to both datasets (**Figure 5E**, **Supplementary Table S7** lists the names of each gene). An analysis of biological functional annotation was conducted on the 86 genes using Metascape. The genes associated with innate immune response, cytokine signaling in the immune system, positive regulation of immune response, and response to type II IFN exhibited significant alterations (**Figure 5F**, **Table 2**). Using the Venn diagram, the genes uniquely expressed in 6-M2d or BAY-M2d were also analyzed in the two donors, or the genes shared between 27-Mac and 6-M2d. In 6-M2d, only nine genes (IL7R, RND3, TNFSF10C, MMP7, SMOX, SLAM1, METIL7B, LINCO1678, and CR1) were identified as unique DEGs (**Supplementary Figure S7A**), whereas in BAY-M2d, none of the genes were commonly expressed (**Supplementary Figure S7B**). The common genes in 27-Mac and 6-M2d were only three (JAK3, PTX3, and GAS6) (**Supplementary Figure S7C**). Within the nine common 6-M2d genes, IL7R, CR1, and SLAMF1 were associated with negative regulation of adaptive immune response, but other genes were not classified due to low gene number.

**Table 1:** The number of DEGs^1^ among different cell types.

Donor 1				
	1	2	3	4
	M2	27-Mac	6-M2d	BAY-M2d
M2				
27-Mac	619			
6-M2d	542	483		
BAY-M2d	380	287	101	

Donor 2				
	5	6	7	8
	M2	27-Mac	6-M2d	BAY-M2d
M2				
27-Mac	348			
6-M2d	36	356		
BAY-M2d	5	253	42	

^1^Genes that were significantly up- or downregulated in expression by more than threefold (*p* < 0.05) in each cell type compared to the reference cells were selected. The total number of selected genes is shown.

**Figure 6:**
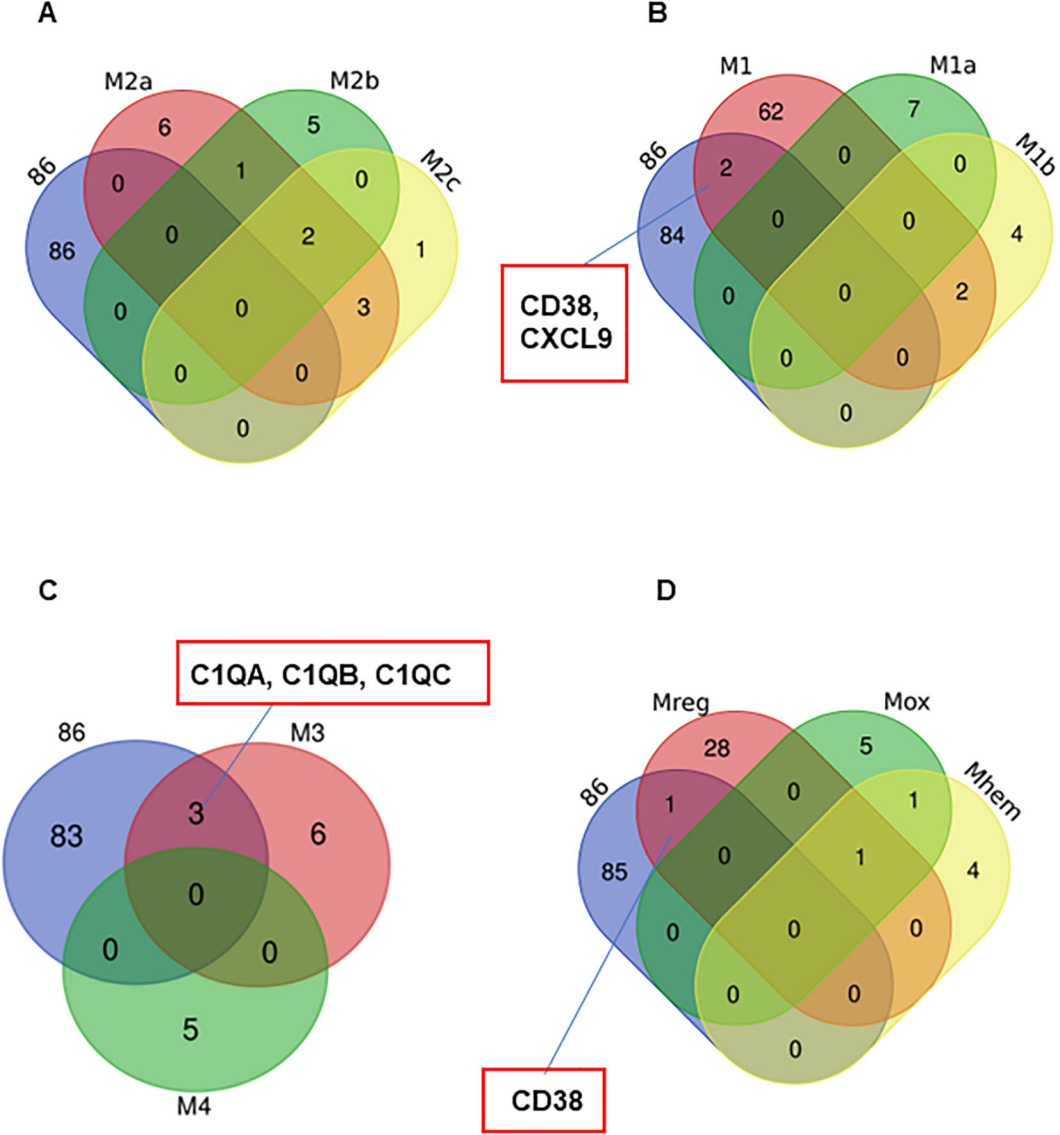
Characterization of the 86 common genes in 27-Mac with other subsets of macrophages. Venn diagram analyses were employed to determine the degree of similarity between the 27-Mac and the macrophage subset signature genes (**Supplementary Table S8**). **(A)** 27-Mac vs. M2a subsets (M2a, M2b, and M2c). **(B)** 27-Mac vs. M1 subset (M1, M1a, and M1b). **(C)** 27-Mac vs. M3 and M4. **(D)** 27-Mac vs. Mreg, Mox, and Mherm.

**Figure 7:**
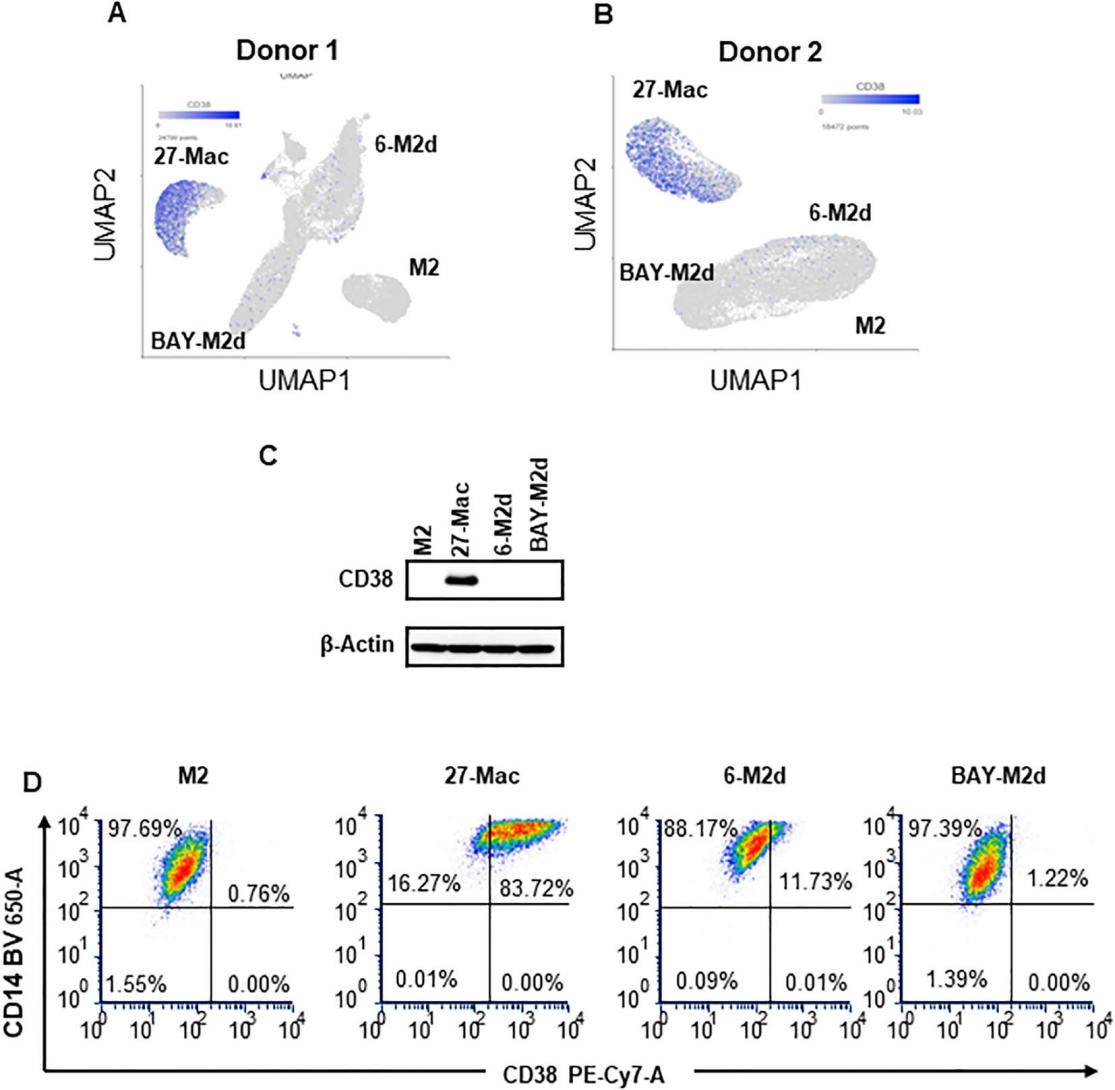
Characterization of CD38 expression. **(A, B)** The expression of the CD38 gene in M2, 27-Mac, 6-M2d, and BAY-M2d of Donors 1 **(A)** and Donor 2 **(B)** is illustrated using UMAP. The blue dots indicate cells that are expressing *CD38*. **(C)** In order to ascertain the expression of CD38 protein on macrophages, monocytes were obtained from donors who were independent of Donor 1 and Donor 2, subsequently differentiated into M2 macrophages, and then polarized using IL-27, IL-6, or BAY60-6583, as described in the Materials and Methods. Each polarized cell was subjected to Western blotting analysis using anti-CD38 or anti-β-actin as an internal control. Representative results from two independent assays are presented. **(D)** CD38 expression on cell surfaces of M2 macrophages, 27-Mac, 6-M2d, and BAY-M2d was analyzed using flow cytometry as described in the Materials and Methods. Representative results from three independent assays are presented. UMAP, Uniform Manifold Approximation and Projection.

The results from UMAP (**Figures 4A, B**) and Venn diagram (**Figures 5C, D**) analyses illustrated that the gene profile in 27-Mac was different from that in 6-M2d or BAY-M2d. Therefore, we hypothesized that 27-Mac may be a new subset of M2 macrophages and compared the 86 genes with markers of other M2 subsets (M2a, M2b, and M2c) (98, 99) (cell marker genes for each subset are listed in **Supplementary Table S8**). The results showed that none of the 86 genes overlapped with the M2a, M2b, and M2c markers (**Figure 6A**). IL-27 may convert M2 into M1-like macrophages (46, 100), M1 subtypes M1a and M1b (101, 102), or recently identified novel macrophage subsets (M3, M4, Mreg, Mhem, and Mox) (103); therefore, the 86 unique genes were cross-analyzed. Venn diagram analysis showed that, of the 86 genes, the expression of *CD38* and *CXCL9* was shared with that in M1 macrophages (**Figure 6B**), and *C1QA*, *C1QB*, and *C1QC* were commonly expressed in M3 macrophages (**Figure 6C**). Comparison of the 86 genes with those of Mreg, Mhem, and Mox indicated that only *CD38* expression was shared with those of Mreg; however, none of the other genes were commonly expressed (**Figure 6D**), suggesting that 27-Mac is an M1-, M3-, or Mreg-like subset in the context of gene expression. Taken together, only *CD38* was associated with cell surface markers on 27-Mac, and the others were secretary proteins; therefore, we assessed the distribution of CD38-expressing cells using UMAP. As illustrated in **Figures 7A** and **B**, CD38 was expressed in 27-Mac, and the expression of *CD38* mRNA in Donors 1 and 2 was observed in 51.6% and 37.2% of 27-Mac, respectively. WB analysis demonstrated that CD38 was detected in only 27-Mac (**Figure 7C**), and fluorescence-activated cell sorting (FACS) analysis revealed nearly 80% of cells as CD38-positive cells (**Figure 7D**, **Table 2**). The expression of mean fluorescence intensity increased 6.2-fold in 27-Mac compared to that in M2 macrophages (**Table 2**). ELISA results showed that the levels of C1Q and CXCL9 in culture supernatants of 27-Mac were 10.4 ± 1.7 and 19,000 ± 3,100 pg/mL, respectively (**Table 2**).

**Table 2:** Quantification of biomarkers.

Biomarkers	M2 macrophages	27-Mac	F.D.^1^	*p*-Value
CD38^2^				
Population (%)	12.3 ± 7.8 (n = 3)^3^	76.8 ± 4.2 (n = 3)	6.2	0.0019
MFI ^4^	12.7 ± 4.8 (n = 3)	835 ± 225 (n = 3)	65.7	0.0217
C1Q (ng/mL) ^5^	N.D ^6^	10.4 ± 1.7 (n = 5)	N/A	N/A
CXCL9 (pg/mL) ^5^	3.2 ± 7.0 (n = 5)	19,818.5 ± 3,137 (n = 5)	62,443	0.0002
IFN-α (pg/mL)^5^	1.94 ± 0.19 (n = 5)	1.69 ± 0.11 (n = 5)	0.87	0.2878
IFN-β (pg/mL)^5^	N.D ^7^	N.D. ^7^	N/A	N/A
WSX (%)^8^	16.8 ± 2.7 (n = 3)^3^	N/A		
gp130 (%)^8^	42.8 ± 5.5 (n = 3)	N/A		
WSX1/gp130 (%)^8^	3.1 ± 1.5 (n = 3)	N/A		

FACS, fluorescence-activated cell sorting.

^1^Fold difference. Values in 27-Mac were compared to those in M2 macrophages (27-Mac/M2).

^2^CD38 expression was assessed by fluorescence-activated cell sorting (FACS) as dissected in the Materials and Methods. Population of CD14 (+)/CD38 (+) is shown in the table.

^3^Data indicate means ± SE.

^4^Mean fluorescence intensity in histogram.

^5^Protein amounts in culture supernatants after polarization were quantified using ELISA kit.

^6^Not detected; the values were below the detection limit (0.9 ng/mL).

^7^Not detected; the values were below the detection limit (1.2 pg/mL).

^8^Percentages of the cells expressing WSX or gp130 proteins on unpolarized M2 cells.

### 3.6 Expression and distribution of interferon-stimulated genes and host factor genes in 27-Mac

**Figure 8:**
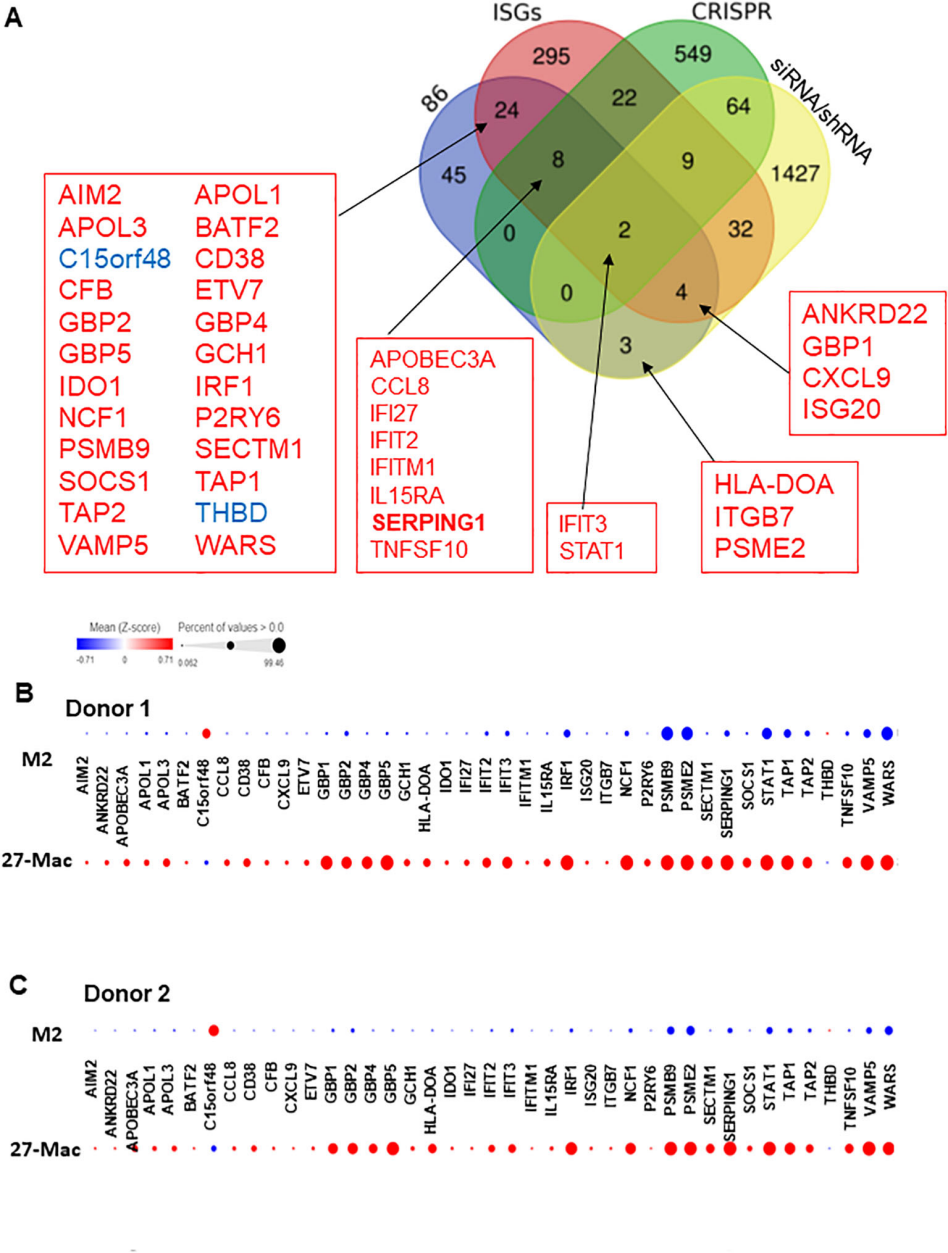
Identification of host factors associated with HIV inhibition in the 86 common genes. **(A)** Venn diagram analysis was conducted using the 86 common genes and a total of 2,439 genes of host factor identified from CRISPR, siRNA, shRNA library screening, and ISGs (**Supplementary Table S9**). **(B, C)** To demonstrate the proportion of cells expressing each of the 41 genes in M2 macrophages and 27-Mac, a bubble plot analysis was performed. The results demonstrate the comparative gene expression between M2 macrophages and 27-Mac from Donor 1 **(B)** or Donor 2 **(C)**. The size of the bubbles is proportional to the percentage of cells in each sample that are expressing the gene of interest. The color intensity is proportional to the relative scaled (Z-score) gene expression within each sample. The relative expression levels are indicated by color, with higher expression levels represented by red and lower expression levels represented by blue. ISGs, interferon-stimulated genes.

IL-27 treatment induces multiple interferon-stimulated genes (ISGs) in macrophages (26, 31, 32, 72) and regulates the expression of multiple host factors (104–106), which are involved in HIV replication and other viral infections. However, the mechanisms of gene expression and gene distribution are not well understood. To identify the genes that play a pivotal role behind the anti-HIV effect of 27-Mac, 86 commonly regulated genes in 27-Mac (**Figure 8A**) were cross-referenced with the 2,120 genes listed in databases of host dependency factors (HDFs) and antiviral genes identified through small interfering RNA, short hairpin RNA, or CRISPR library screening (104, 107–121) and 396 ISGs (**Supplementary Table S9**). The results indicated that 41 genes overlapped with the databases (**Figure 8A**): 38 genes belonged to ISGs. It has been reported that IL-27-mediated cellular responses are associated with type I IFN production (122), but this effect was cell type dependent (19, 72, 123). To elucidate whether type I IFNs were produced during polarization, all subtypes of IFN-α and IFN-β were quantified using ELISA kits with culture supernatants from cells treated with or without IL-27. A low level of IFN-α production was detected from M2 macrophages, the production from 27-Mac was merely decreased (*p* > 0.05) (**Table 2**), and IFN-β production was not detected from both cell types (**Table 2**). These results suggested that the induction of ISGs may not be associated with IFN production. Since IL-27 induced STAT1 activation as did IFN-γ (26, 124), IL-27 may directly induce the ISGs without IFN production.

**Figure 9:**
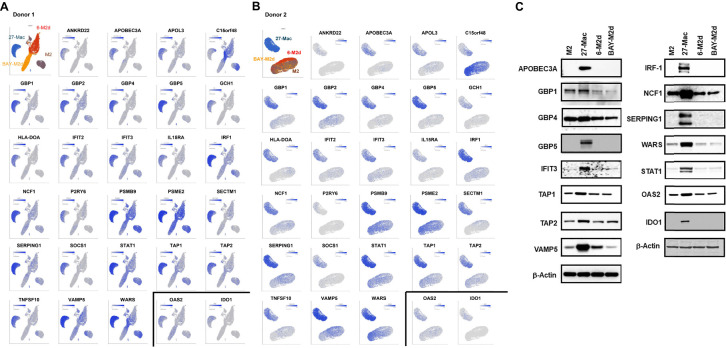
Distribution of antiviral genes in each subset of macrophages. **(A, B)** The genes expressed in >40% of cells in either 27-Mac or M2 macrophages were selected from the 41 antiviral genes (**Figure 8**). The results of 27 genes in Donor 1 **(A)** and Donor 2 **(B)** were analyzed to define gene distribution and population using UMAP. The genes of interest are colored blue. **(C)** To ascertain the expression of antiviral proteins in macrophage subsets, monocytes were obtained from two independent donors and subsequently differentiated into M2 macrophages. M2 macrophages were then polarized into 27-Mac, 6-M2d, and BAY-M2d as described in the Materials and Methods. Each polarized cell was subjected to Western blotting analysis using total of 16 antibodies or anti-β-actin as an internal control. Representative results from two independent assays are presented. UMAP, Uniform Manifold Approximation and Projection.

Of the 38 ISGs, 14 genes were identified as HDFs or antiviral proteins (**Figure 8A**). STAT1 and IFIT3 were the DEGs that overlapped with all four datasets. The Z-scores of each of the 41 genes were calculated using relative expression across all cells of M2 macrophages and 27-Mac of Donor 1 (**Figure 8B**) and Donor 2 (**Figure 8C**), and the results are presented as bubble plots; the size of bubbles represent the percentage of expressing cells. The expression of two ISGs (*C15or48* and *THBD*) was decreased in 27-Mac compared to that in M2 macrophages; other gene expression was increased in 27-Mac (**Supplementary Table S10**). Of the 41 genes, genes expressed in >40% of either 27-Mac or M2 macrophages were selected (**Supplementary Table S10**). The resulting 27 genes were further analyzed to define gene distribution and population using UMAP (**Figures 9A, B**). In addition to the 27 genes, two known antiviral ISGs—*OAS2* (125, 126) and *IDO1* (119)—were also included, although the genes exhibited lower levels in 27-Mac compared to other genes and were, therefore, filtered out during the selection process; the proteins encoded in these genes are known to exhibit broad antiviral activity. Using a total of 29 genes, UMAP analyses were conducted. The results illustrated that the expression of ISGs and host factors exhibited a donor and individual cell-dependent manner even in the polarized 27-Mac. Population analysis with UMAP demonstrated that 40% to 70% of M2 macrophages of Donor 1 and Donor 2 expressed *PSMB9*, *PSME2*, *STAT1*, *TAP1*, *VAMP5*, or *WARS* (**Supplementary Table S10**). After polarization with IL-27, the population of cells expressing those six genes was increased to more than 95% (**Supplementary Table S10**). On average, the expression of those genes in 27-Mac was increased by 1.4 ± 0.2-fold in Donor 1 (*p* = 0.0036) and 2.1 ± 0.6-fold in Donor 2 (*p* < 0.0001). In contrast, the expression of 13 genes (*APOL1*, *APOL3*, *GBP1*, *GBP2*, *GBP5*, *IFIT2*, *IFIT3*, *IRF1*, *NCF1*, *SERPING1*, *SOCS1*, *TAP2*, or *TNSF10*) in M2 macrophages and 27-Mac was 6%–33% and 30%–95%, respectively (**Supplementary Table S10**). Consequently, the mean population of 27-Mac expressing these genes was significantly increased by 4.7 ± 0.80-fold (*p* < 0.0001) in Donor 1 and 5.8 ± 1.00-fold (*p* < 0.0001) in Donor 2 compared to that of M2 macrophages. The remaining 20 genes (*AIM2*, *ANKRD22*, *APOBEC3A*, *BATF2*, *CCL8*, *CD38*, *CFB8*, *CXCL9*, *ETV7*, *GBP4*, *GCH1*, *HLA-DOA*, *IDO1*, *IRF27*, *IFITM1*, *IL15RA*, *ISG20*, *ITGB7*, *PSRY6*, and *SECTM1*) were expressed in less than 5% of M2 macrophages of both donors. The percentages of 27-Mac expressing these genes were significantly increased by 149.3 ± 45.0-fold (n = 20) in Donor 1 (*p* < 0.0001) and 35.6 ± 8.7-fold in Donor 2 (*p* < 0.0001) (**Supplementary Table S10**). To determine whether the products of the upregulated genes were expressed in the cells, the protein expression of randomly selected genes was validated by WB. A total of 17 proteins including CD38 (**Figure 5C**) were predominantly or uniquely induced in 27-Mac (**Figure 9C**).

### 3.7 Impact of the expression of WSX1, gp130, CD4, and CCR5 genes in 27-Mac

**Figure 10:**
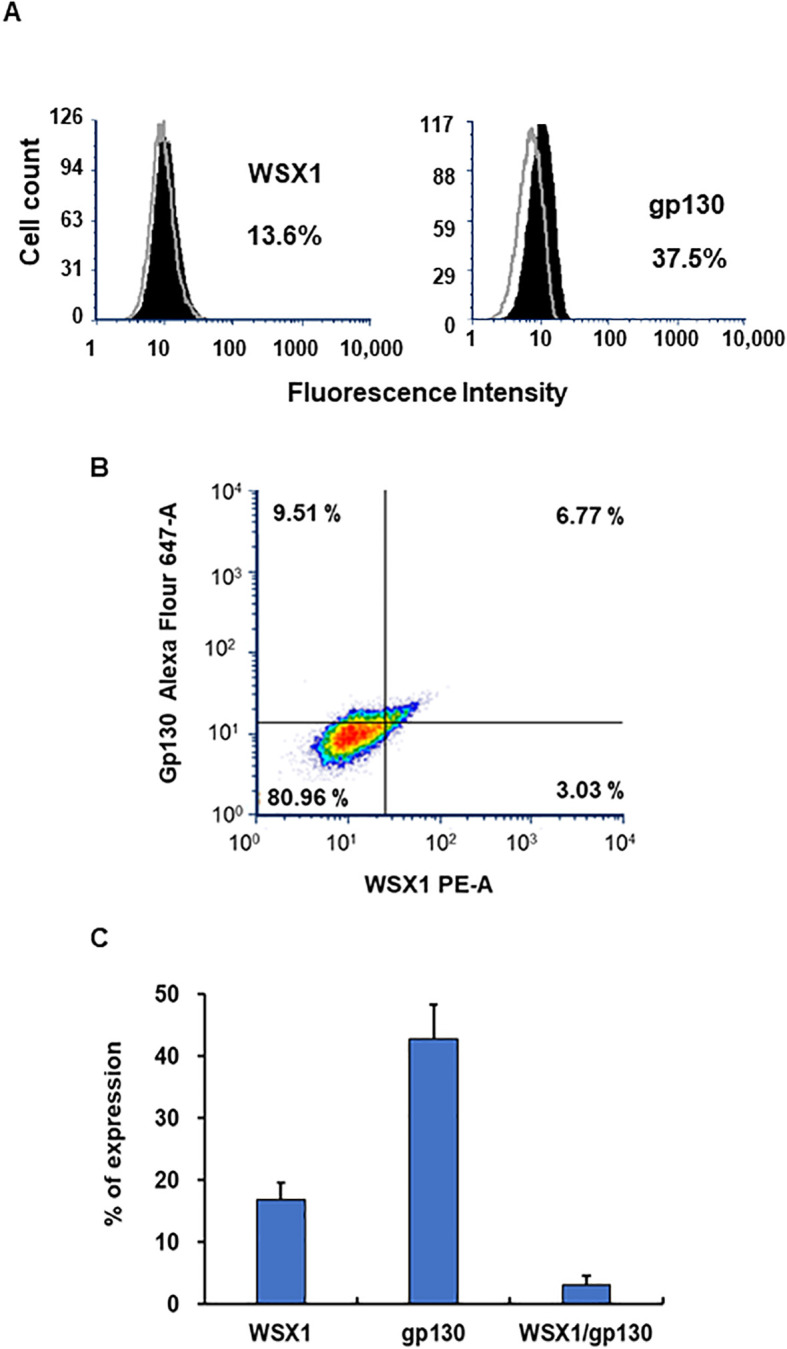
Detection of WSX and gp130 on M2 macrophages using FACS. The surface expression of WSX1 and gp130 on M2 macrophages was assessed by flow cytometry as described in the Materials and Methods. **(A)** The left and right panels show WSX1 and gp130 (CD130) staining, respectively. The staining pattern of isotype control antibodies is shown in gray line, and black indicates the protein of interest. The x-axis and y-axis show fluorescence intensity and cell counts, respectively. The percentages in the panels indicate the percentage of cells expressing WSX1 or gp130 in the samples. Data are representative of three independent experiments with similar outcomes. **(B)** Flow cytometric analysis of M2 macrophages showing the expression of WSX1 and gp130 (CD130) from one donor M2 macrophages. **(C)** FACS analysis for WSX1 and gp130 expression on M2 cells was performed using three independent donor cells. Results indicated means ± SE (n = 3). FACS, fluorescence-activated cell sorting.

IL27R consists of WSX1 (also known as TCCR) and gp130. The expression of WSX1 in monocytes and macrophages is lower than that in T cells or NK cells (4, 124, 127, 128). To define the population of cells expressing WSX1 and gp130 in M2 macrophages prior to polarization, flow cytometric analysis was performed. **Figures 10A** and **B** show results from one of the donors. Consistent with others, the protein expression of WSX1 was low. FACS assays using three independent donors illustrated that WSX1 and gp130 protein expressed on 16.8% ± 2.7% (n = 3) and 42.8% ± 5.5% (n = 3) of M2 macrophages, respectively (**Figure 10C**), and the cells expressing both proteins were 3.1% ± 1.5% (n = 3) (**Figure 10C**). The scRNA-Seq analysis revealed that the expression of both *WSX1* and *GP130* (*WSX1^+^*/*GP130^+^*) in M2 was 7.9% in Donor 1 and 4.6% in Donor 2, while both genes were detected in 5.9% of Donor 1 and 1.6% of Donor 2 of 27-Mac (**Table 2**), indicating that the polarization to 27-Mac resulted in a decrease rather than an increase in the population of WSX1/gp130-expressing cells.

**Figure 11:**
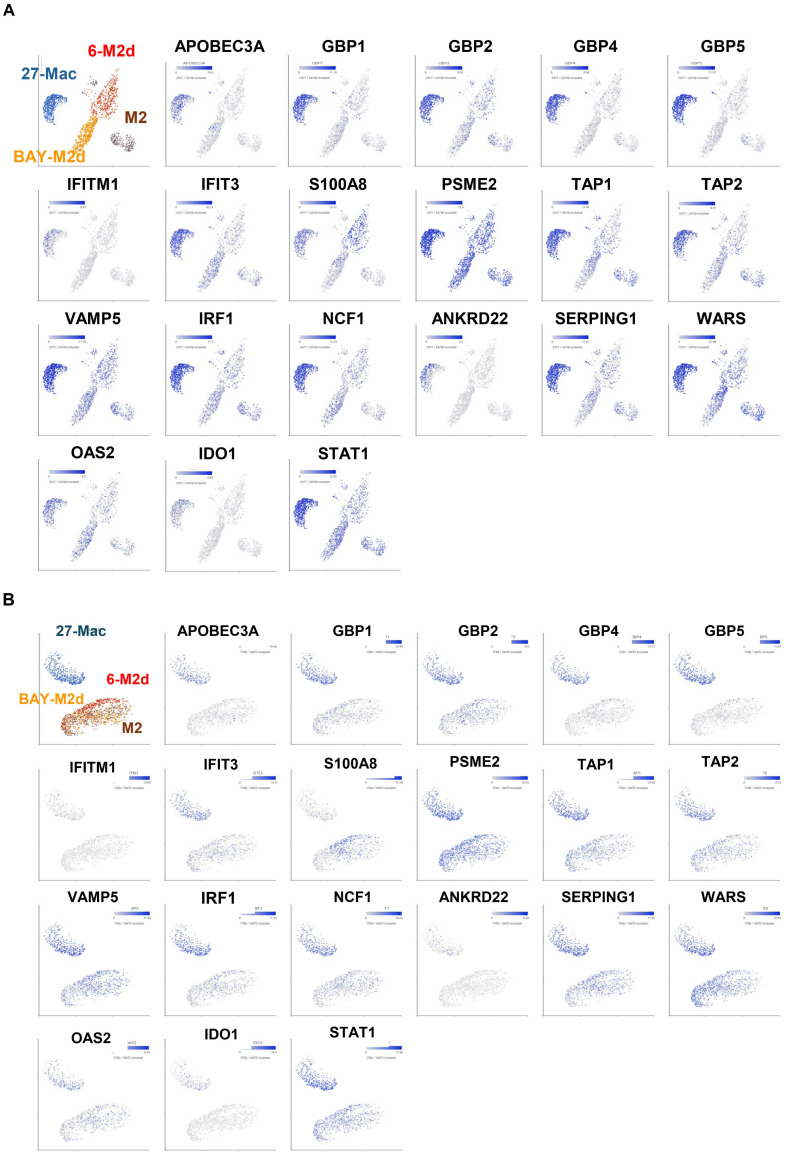
Distribution of antiviral genes in *CD4*(*+*) and *CCD5*(*+*) each macrophage. Cells expressing both *CD4* and *CCR5* (the cutoff is 4 or above for expression) were selected from whole M2 macrophages, 6-M2d, BAY-M2d, and 27-Mac of Donor 1 **(A)** and Donor 2 **(B)**. UMAP analysis for CD4(+)/CCR5(+) cells for each subtype was conducted by extracting them from the UMAP for entire subtype (**Figures 7A, B**). The UMAPs for genes of interest were colored blue to demonstrate the expression level. UMAP, Uniform Manifold Approximation and Projection.

HIVAD8 is an R5 tropic virus that infects cells through CD4 and CCR5 (129). As shown in **Figure 1A**, HIVAD8 replication was suppressed in 27-Mac. To predict the mechanism of inhibition in CD4+CCR5+ cells, the distribution of antiviral genes in 27-Mac expressing both *CD4* and *CCR5* was analyzed using UMAP (CD4+CCR5+ population in 27-Mac in Donor 1 and Donor 2 was 11.2% and 7.6%, respectively) (**Supplementary Table S11**). In the analysis, a total of 20 genes [15 genes were subjected in **Figure 9**, and GBP2, IFITM1, PSME2, ANKRD22, and S100A8 (130)] were analyzed. The UMAP showed that each gene expression was not clustered in both donors (**Figures 11A, B**). A population analysis of cells expressing the antiviral genes revealed that nine out of the 20 genes in Donor 1 and 11 out of the 20 genes in Donor 2 were expressed in less than 20% of M2. However, in 27-Mac, these genes were expressed in nearly 50% (17.7%–98.0%) of Donor 1 (average 52%) and 6.9%–96.4% of Donor 2 (average 47.6%) (**Supplementary Table S12**). The mean fold increases in the cells expressing the genes were 19 ± 7.2-fold (*p* < 0.001) in Donor 1 and 9.9 ± 2.6-fold (*p* < 0.01) in Donor 2, compared to those in M2 macrophages (**Supplementary Table S12**). The expression level of each gene differed among individual cells. However, the distribution of the genes among *CD4*(+)/*CCR5*(+) cells was similar to that observed in the entered 27-Mac, with no clustering evident among the two donors. This finding suggests that each HIV-susceptible cell in 27-Mac expresses multiple antiviral genes, but not the same expression profiles in all cells. Thus, the anti-HIV effect observed in 27-Mac appears to be regulated by the cooperative action of the products of multiple genes, and the mechanism of the anti-HIV effect in 27-Mac may differ in each cell.

## 4 Discussion

IL-27 possesses antiviral properties and inhibits the replication of RNA and DNA viruses in several cell types. We have previously noticed that 27-Mac exhibits resistance to HIV infection (32); however, the mechanism underlying this inhibition is not clearly understood. Given that IL-27 and IL-6 share gp130 and that IL-6 polarizes M2 macrophages to M2d (6-M2d), we postulated that 27-Mac may be M2d-like macrophages. We found that 27-Mac has phenotypical differences from other M2d cells, especially ROS-inducing activity, and the expression of 86 genes was differentially regulated in 27-Mac. The 86 genes contained 38 ISGs. The 86 genes were involved in innate immune responses and cellular activation. As we previously demonstrated (32), the enhanced ROS activity appeared to be the result of an increase in the expression of the NCF-1 (p47phox) gene among the 86 genes. The phagocytic activity of 27-Mac was comparable to that of 6-M2d and BAY-M2d. Therefore, DEGs in 27-Mac may not be involved in its function. Although IL-27 shares gp130 with IL-6, IL-27 stimulation induced STAT1 activation in addition to STAT3 activation, suggesting that 86 gene activation may be mediated by STAT1 or a combined STAT1 and STAT3 pathway; this pathway may contribute to the anti-HIV effect in 27-Mac.

To determine whether 27-Mac is a subtype that has converted into another subtype, we compared the 86 genes in 27-Mac with signature gene profiles of other macrophage subtypes (M1, M2a, M2b, M2c, M3, M4, Mreg, Mhem, and Mox), and *CD38*, *CXCL9*, and *C1Q* were identified as common genes shared with other subtypes. The majority of 27-Mac expressed CD38 protein on the cell surface, and CXCL9 and C1Q proteins were significantly secreted by 27-Mac. CD38 is a biomarker of M1 and Mreg, and C1Q is a biomarker of M3 macrophages. It appears that 27-Mac may be converted from M2 macrophages to M1- or Mreg/M3-like subset. To further elucidate the function of the 27-Mac, a comparative study using 27-Mac and M1 is necessary; currently, the comparative study is in progress.

CD38 is a multifunctional transmembrane ectoenzyme identified as a marker of T- and B-cell activation and macrophages (131). It functions as a cyclic adenosine diphosphate-ribose (cADPR) hydrolase, catalyzing the degradation of cADPR to ADPR and converting nicotinamide adenine dinucleotide (NAD) to cADPR (131, 132). cADPR increases the concentration of intracellular calcium (Ca^2+^) via calcium channels (133, 134); thus, 27-Mac may regulate Ca^2+^-dependent cell signaling such as Ca^2+^-dependent kinases (135, 136) and modulate Ca^2+^-dependent mitochondria function (137), subsequently cytokine production and differentiation (138). The increase in intracellular Ca^2+^ may also activate Ca^2+^-dependent transcription factors, such as calcium-responsive transcription factor (CaRF) (139), cyclic adenosine monophosphate response element binding protein (CREB) (140), or nuclear factor of activated T cells (NFATA) (141). Those factors may be involved in the induction of the 86 unique gene expression in 27-Mac. cADPR negatively regulates ISG induction (142), suggesting that CD38 in 27-Mac may be involved in the differential expression of ISGs. CD38 also plays a role in cell adhesion (132). CD38 can function as a receptor with the ability to bind CD31 (PCAM-1). CD31 is expressed in naïve T-cell population and also macrophages (143, 144); thus, the interaction of CD38 and CD31 between macrophages may regulate the 27-Mac cellular function. A correlation between CD38 and HIV pathogenesis has been reported (145–147), although these reports focus on CD38 function in T cells rather than macrophages (147). Consequently, further studies are necessary to elucidate the role of CD38 in the cellular function of 27-Mac and its involvement in HIV suppression.

CXCL9, also known as a monokine induced by IFN-γ (MIG), is produced by IFN-γ, but not by IFN-α/β (148, 149). In the current study, unpolarized M2 cells produced IFN-γ, which is reported to be produced from M1 but not M2 (150). Since unpolarized cells were cultured in the presence of FBS, the spontaneous production of IFN-γ may be regulated by supplemented serum, and the production of IFN-γ was suppressed in 27-Mac; thus, the induction of CXCL9 may be IFN-γ independent. To further characterize 27-Mac, a comparative study using 27-Mac and IFN-γ-treated M2 cells is necessary. CXCL9 acts as a chemoattractant for T cells via its receptor, C-X-C motif chemokine receptor 3 (CXCR3), on T cells. Therefore, 27-Mac may enhance the communication between T cells and macrophages and regulate T-cell function. CD38 is known as a T-cell activation and differentiation marker. In our previous studies, we have not observed the induction in T cells treated with IL-27 (72, 151). Thus, IL-27 may regulate the activation of the CD38 gene in macrophages and T cells in a deferential manner.

The expression of WSX1 (also known as TCCR) in monocytes and macrophages is lower than that in T cells or NK cells (4, 124, 127, 128). The scRNA-Seq analysis using fresh PBMCs demonstrated that approximately 1% of total monocytes in PBMCs expressed both *WSX1* and *GP130* (personal communication, Xuan Li and Tomozumi Imamichi, November 27, 2024, **Supplementary Table S13**). A lower level of detection of *WSX1* or *GP130* may be due to the limited depth of scRNA-Seq; therefore, the value of the expression level may not accurately reflect the absolute value of the expressed proteins or genes. Despite the fact that, in the current study, scRNA-Seq analysis illustrated that 6.25% ± 1.7% (n = 2) of M2 macrophages were *WSX1*(*+*)/*GP130*(*+*) cells, while 3.8% ± 2.2% (n = 2) of 27-Mac expressed *WSX1*(*+*)/*GP130*(*+*). Thus, the polarization of M2 to 27-Mac decreased rather than increased in IL27R-expressing cells; however, 50%–95% of 27-Mac expressed ISGs (*GBP1*, *GBP2*, *GBP4*, *GBP5*, *APOL3*, *IFIT2*, *IFIT3*, *STAT1*, *SERPING1*, *SOCS1*, *TAP1*, *TAP2*, *VAMP5*, and *WARS*).

It has been reported that the induction of ISGs may not be directly induced by IL-27; it may be mediated by uncharacterized soluble factor(s), which are induced by IL-27-responding cells. IL-27-mediated antiviral mechanism is considered IFN-dependent in human serum-induced macrophages (122); therefore, IFNs were considered the soluble factors in 27-Mac in the current study. However, enhancement of any IFN (IFN-α, β, and Λ) genes was not detected in scRNA-Seq analysis, and ELISA results using culture supernatants from during polarization demonstrated that neither IFN-α nor IFN-β was detected. These results implicate that uncharacterized soluble factors, rather than IFNs, may be induced by M2 macrophages responding to IL-27. As IL-27 induced activation of STAT1, some genes are directly activated by IL-27. These factors may trigger polarization and induce the expression of ISGs and antiviral genes, including anti-HIV genes. Since scRNA-Seq demonstrated that each cell expresses multiple different antiviral genes, 27-Mac may suppress broad viral infection including HIV through either a single or a combination of the gene products in an individual cell-dependent and donor-dependent manner; therefore, it cannot be defined by a single mechanism. In the current study, we demonstrated for the first time that the A_2b_AR agonist, BAY60-6583, inhibits HIV replication in macrophages. The precise mechanism of its anti-HIV effect remains unclear; however, the expression of the 17 anti-HIV genes that significantly increased in *CD4*(+)/*CCR5*(+) 27-Mac was not augmented in BAY-M2d (**Supplementary Table S10**); those genes appear not to be involved in the anti-HIV effect in the BAY-M2d. BAY-M2d induced multiple antiviral genes (*TLR7*, *CD74*, *CES1*, *HLA-DOA*, *CD209*, and *CMPL2*), the products of which may be involved in resistance to viral infections.

In the current study, 27-Mac is resistant to HIV. The gene expression profile of 27-Mac was distinct from that of conventional M2d (IL-6 or adenosine-polarized M2 macrophages). In the current study, scRNA-Seq analysis was carried out using two independent donor cells. Although WB, IsoPlexis, and ELISA were conducted using a total of more than 10 independent donors, the sample size may not be sufficient to make our findings in general, especially to demonstrate cell clustering among 27-Mac, 6-M2d, and BAY-M2d. In the current study, taking advantage of scRNA-Seq, we found that 86 genes were uniquely expressed in 27-Mac. Further studies of each gene function may help to understand the specific functional properties of 27-Mac. However, the results of this study provide new insights into the function of IL-27 and the potential existence of a novel subset in macrophages resisting broad viral infection by inducing multiple ISGs in most cells. Although an effective combination of antiretroviral regimens has been developed for HIV infection in clinical therapy, the emergence of multidrug resistance and transmission of drug-resistant HIV strains limit the clinical efficacy. Since IL-27 has been demonstrated to effectively suppress HIV, it may serve as a potential agent for treating multidrug-resistant HIV infection. IL-27 serves as an antiviral reagent against influenza virus, hepatitis C virus, hepatitis B virus, cytomegalovirus coxsackievirus B3, respiratory syncytial virus, dengue virus, chikungunya virus, Zika virus, and Mayaro virus *in vitro* (13, 20–29). Since the expression profiles of antiviral genes are different among cells, understanding the exact mechanism of anti-HIV effects in each infected cell warrants further investigation. Our result supports others indicating that IL-27 induces ISGs and antiviral effects (26, 86). ROS-inducing activity is considered an M1 marker (152), and IL-27 treatment enhances ROS induction in M2 cells; therefore, IL-27-induced polarization may switch M2 into M1 cells like other reagents (153). Although we need further characterization of 27-Mac to determine whether the cells are the converted M1 macrophages, IL-27 may regulate the M1/M2 polarizing ratio, which affects the development of autoimmune disease (154, 155) or tumor (156) and immunoregulation and therapeutics (157). Thus, IL-27 may serve as a potential cytokine-based therapy for various viral infections and autoimmune diseases, and cancer progression.

## Data Availability

The data sets for scRNA-Seq in this study can be found in the Gene Expression Omnibus (GEO) data base, the access number is GSE284567.
