# Supplementary material for: Interleukin-27-polarized HIV-resistant M2 macrophages are a novel subtype of macrophages that express distinct antiviral gene profiles in individual cells: implication for the antiviral effect via different mechanisms in the individual cell-dependent manner
Source: Front Immunol. 2025 Mar 10;16:1550699. doi: 10.3389/fimmu.2025.1550699 (PMC11931227; doi:10.3389/fimmu.2025.1550699)
Supplement: Supplementary file 14 [file Image1.pdf]

Supplemental Figure S1

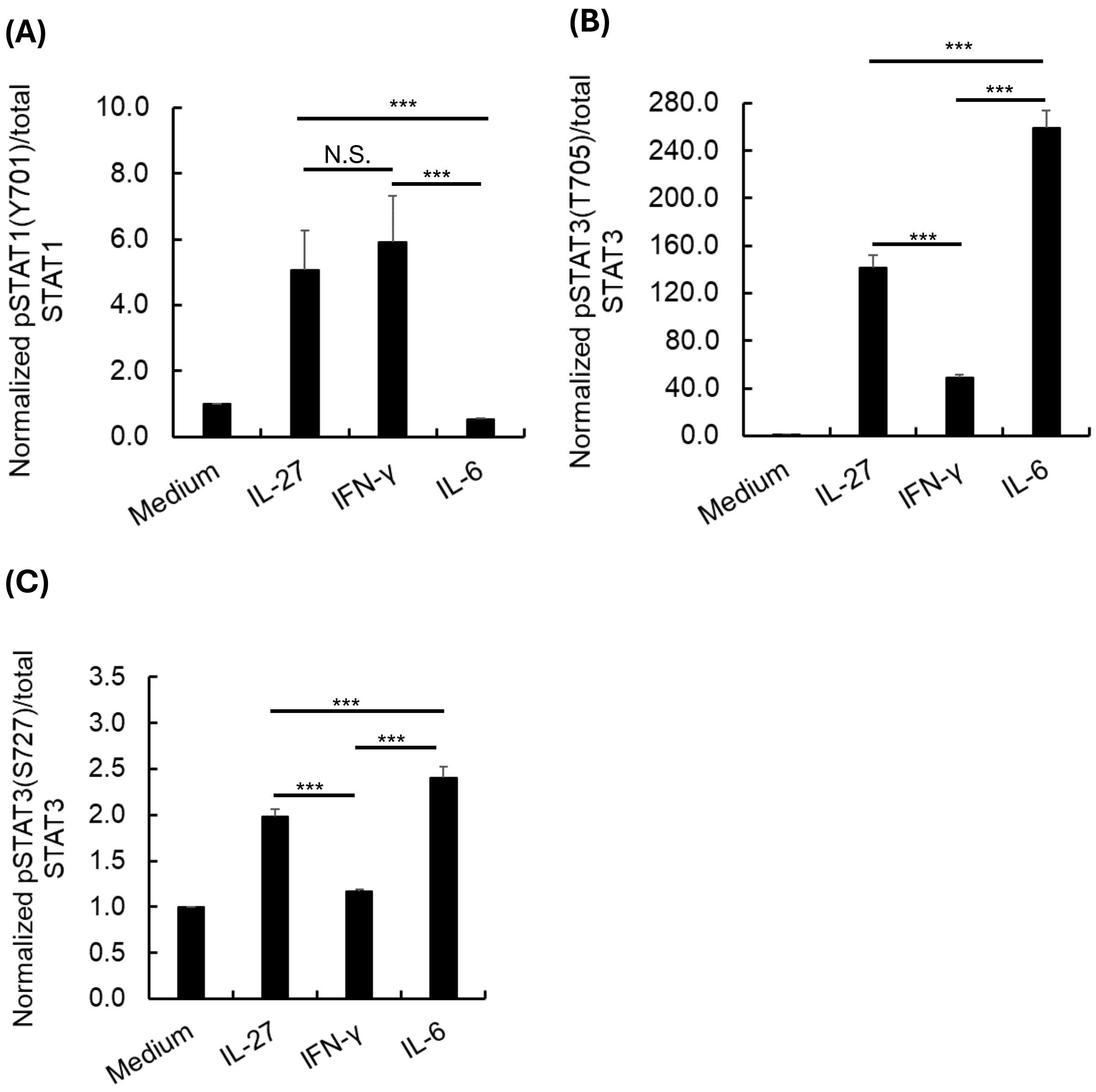

Bands intensity of phosphorylated protein was analyzed by Fiji (Image J). The intensity of the band for (A) pSATAT1 (Y701), (B) pSTAT3(T705), (C) pSTAT3(S727) was normalized by the intensity of total STAT1 and total STAT3, respectively. The densitometry analysis was performed for three independent times, and the data are shown as mean ± SD (n=3).
