# Supplementary figures and images for: Interleukin-27-polarized HIV-resistant M2 macrophages are a novel subtype of macrophages that express distinct antiviral gene profiles in individual cells: implication for the antiviral effect via different mechanisms in the individual cell-dependent manner
Source: Front Immunol. 2025 Mar 10;16:1550699. doi: 10.3389/fimmu.2025.1550699 (PMC11931227; doi:10.3389/fimmu.2025.1550699)

# Supplementary Figure S2

## Donor 1 Cluster 1

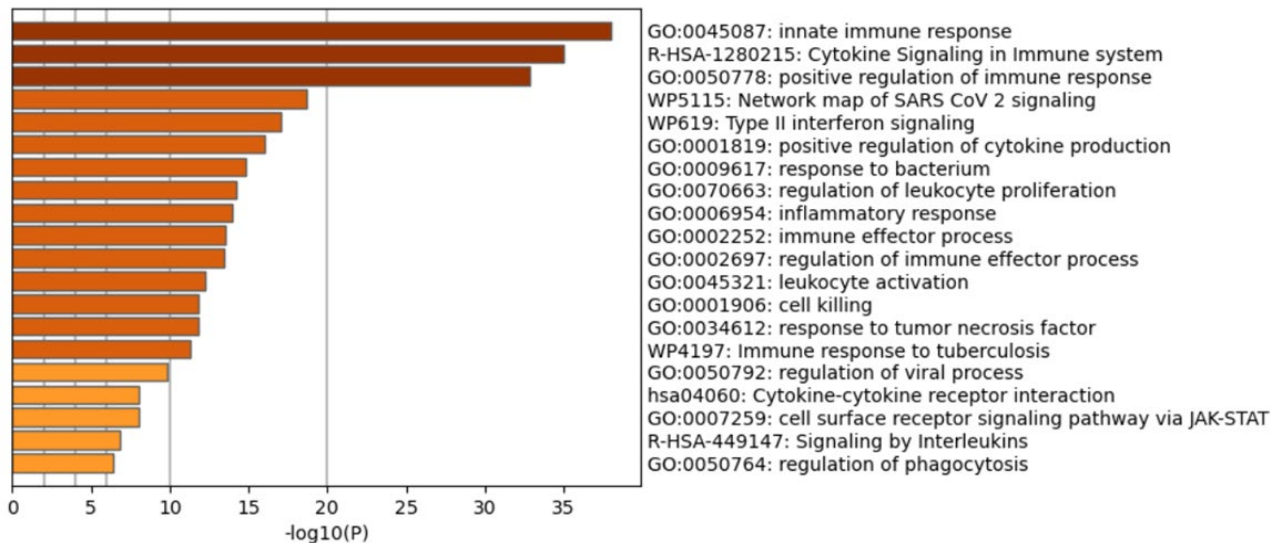

## Donor 1 Cluster 2

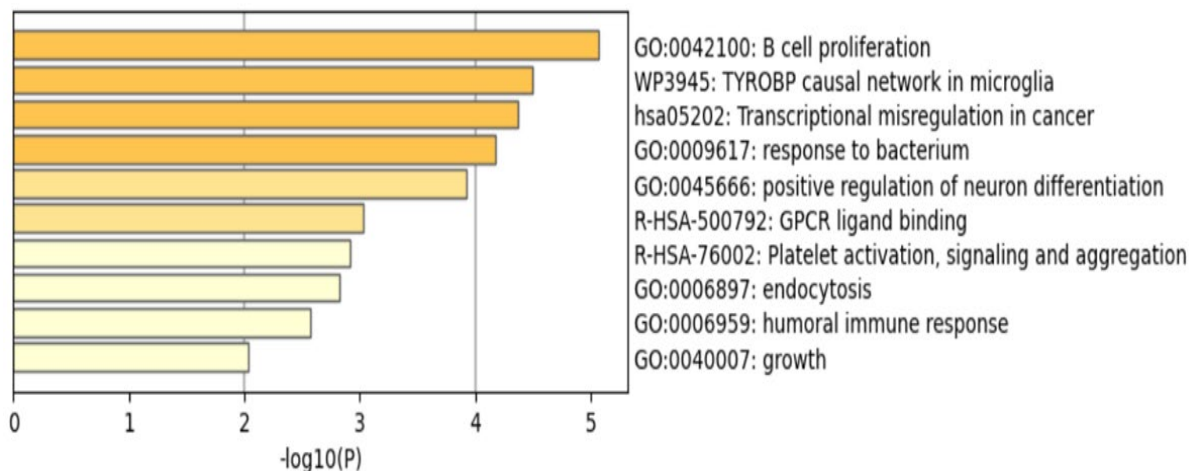

## Donor 1 Cluster 3

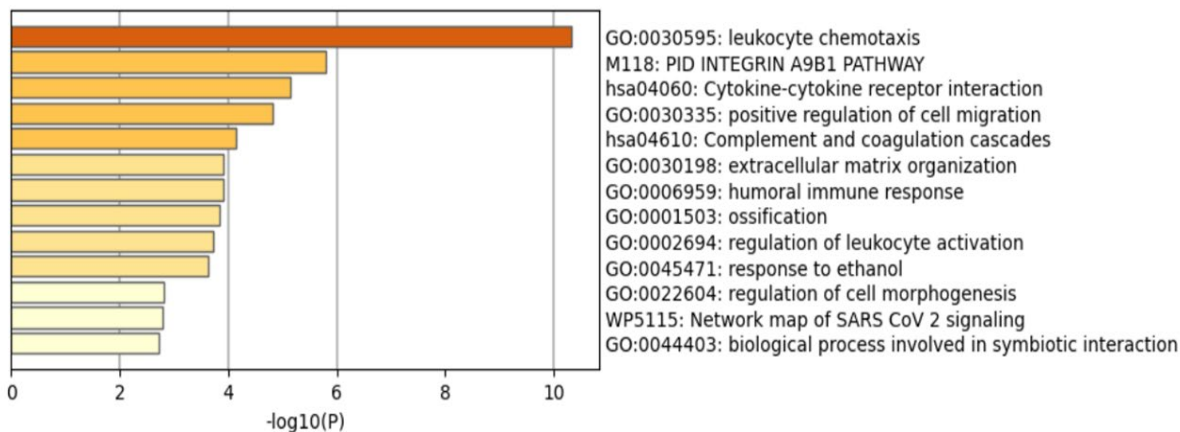

Supplement: Supplementary file 15 [file Image2.pdf]

# Supplementary Figure S3

## Donor 1 Cluster 4

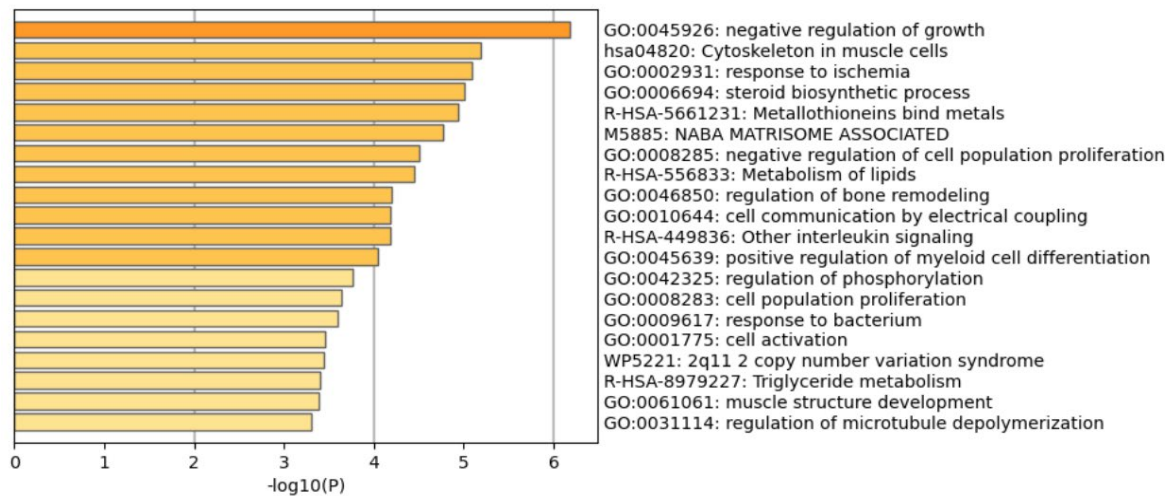

## Donor 1 Cluster 5

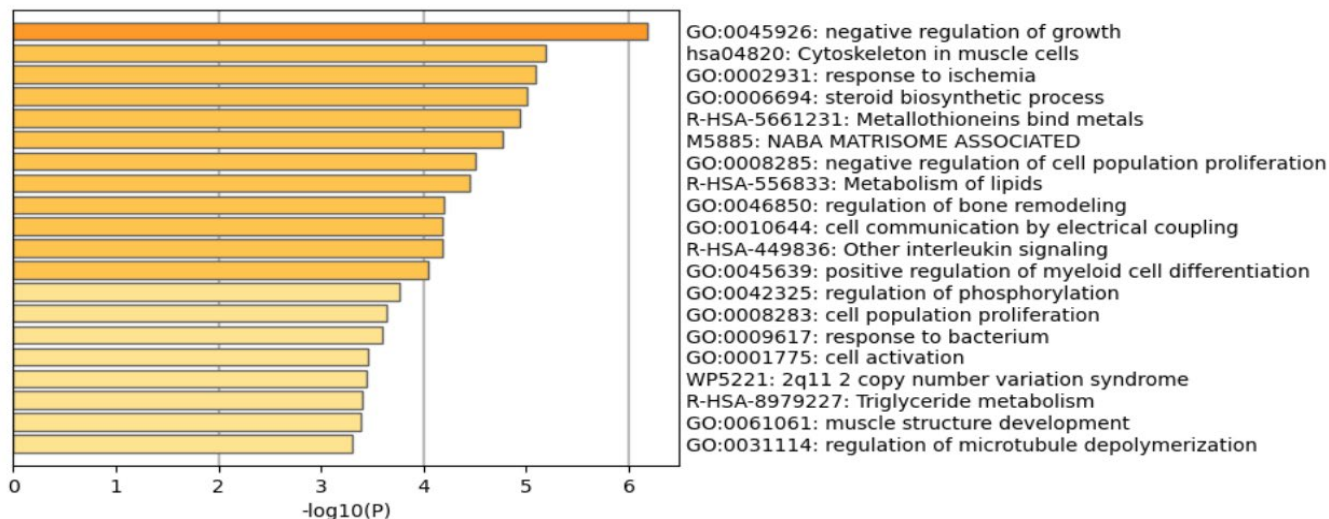

## Donor 1 Cluster 6

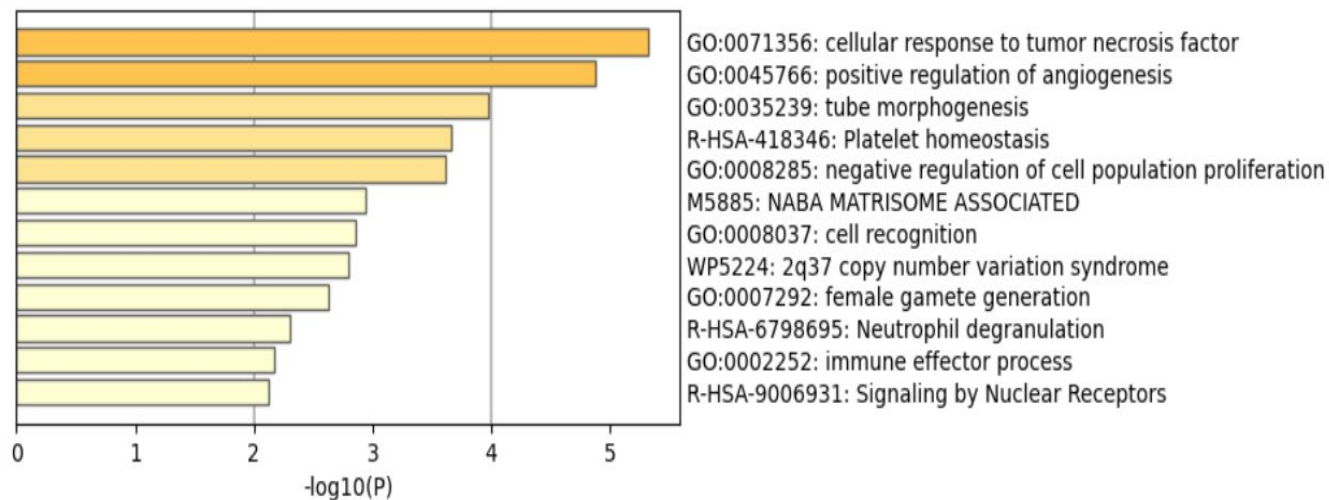

Supplement: Supplementary file 16 [file Image3.pdf]

# Supplementary Figure S4

## Donor 1 Cluster 7

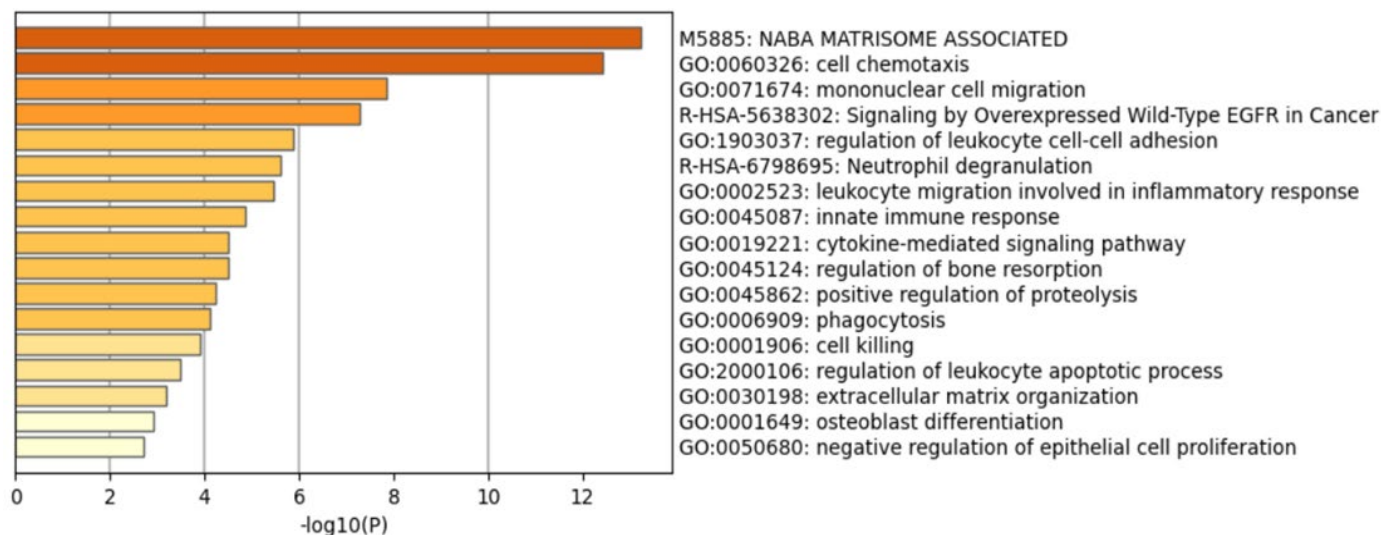

## Donor 1 Cluster 8

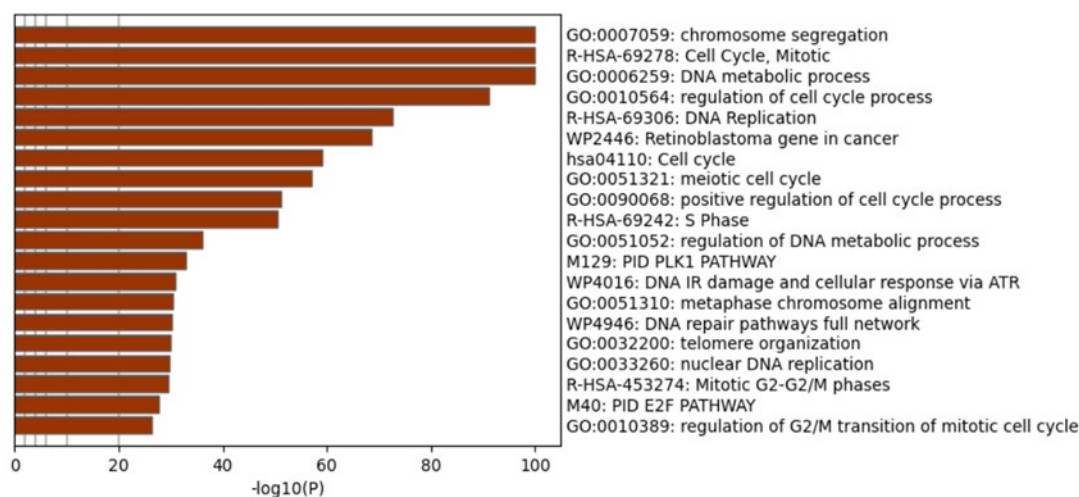

## Donor 1 Cluster 9

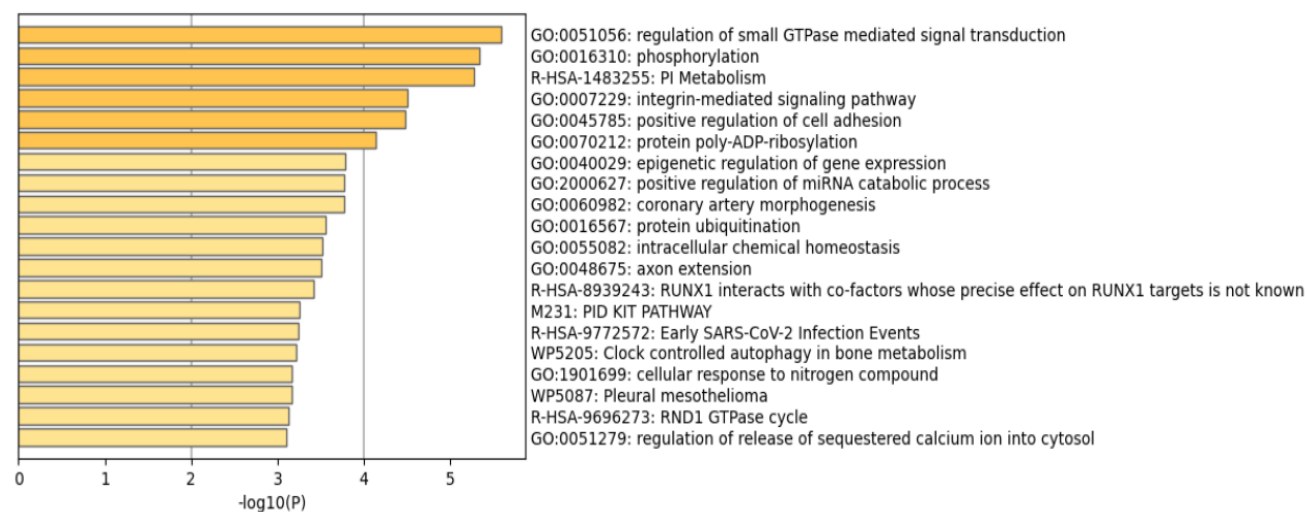

Supplement: Supplementary file 17 [file Image4.pdf]
