# Supplementary material for: Interleukin-27-polarized HIV-resistant M2 macrophages are a novel subtype of macrophages that express distinct antiviral gene profiles in individual cells: implication for the antiviral effect via different mechanisms in the individual cell-dependent manner
Source: Front Immunol. 2025 Mar 10;16:1550699. doi: 10.3389/fimmu.2025.1550699 (PMC11931227; doi:10.3389/fimmu.2025.1550699)

Supplementary Figure S5

Donor 2 Cluster 1

Only two genes (LYPD1 and CAMP) were categorized in this cluster, Functional annotation analysis was not conducted.

Donor 2 Cluster 2

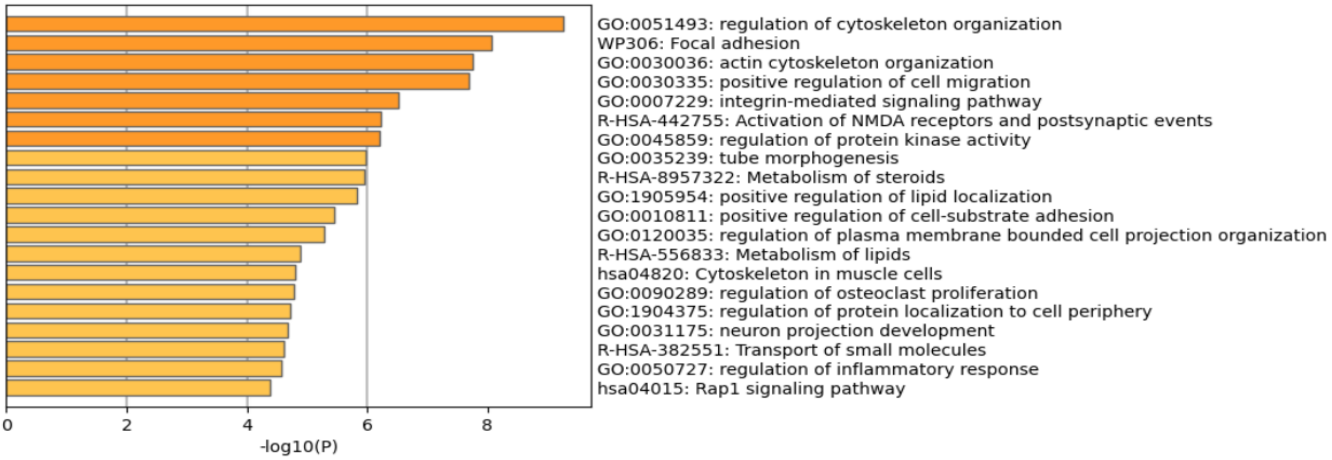

Donor 2 Cluster 3

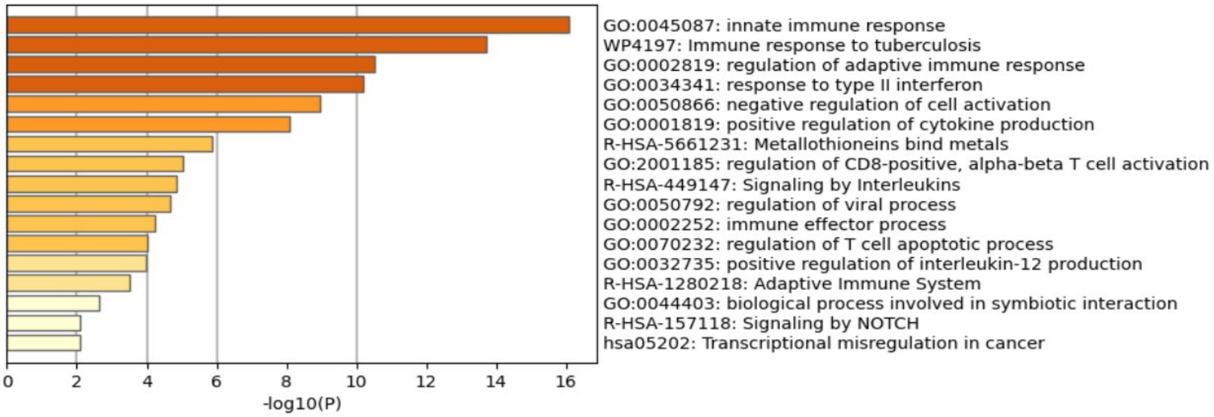

Donor 2 Cluster 4

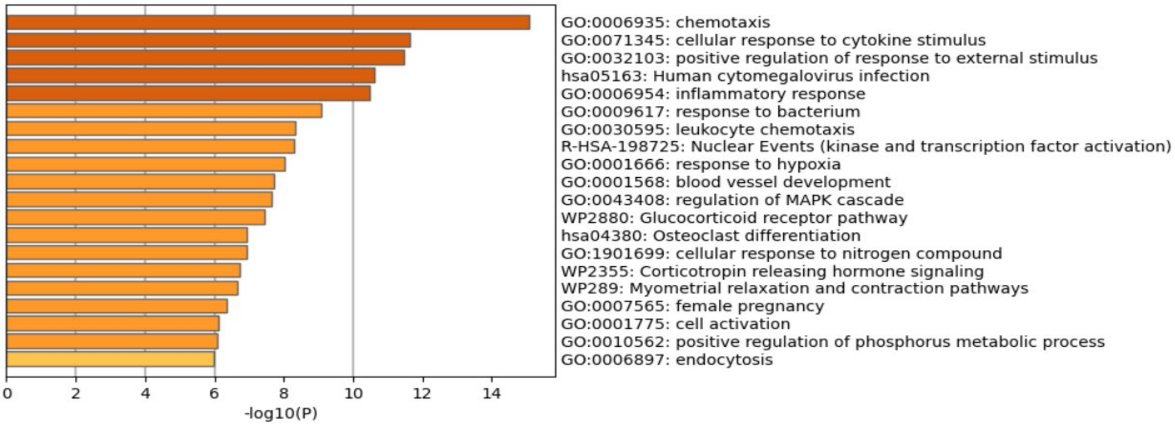

Supplement: Supplementary file 18 [file Image5.pdf]
