# Supplementary material for: Interleukin-27-polarized HIV-resistant M2 macrophages are a novel subtype of macrophages that express distinct antiviral gene profiles in individual cells: implication for the antiviral effect via different mechanisms in the individual cell-dependent manner
Source: Front Immunol. 2025 Mar 10;16:1550699. doi: 10.3389/fimmu.2025.1550699 (PMC11931227; doi:10.3389/fimmu.2025.1550699)
Supplement: Supplementary file 19 [file Image6.pdf]

Supplementary Figure S6

Donor 2 Cluster 5

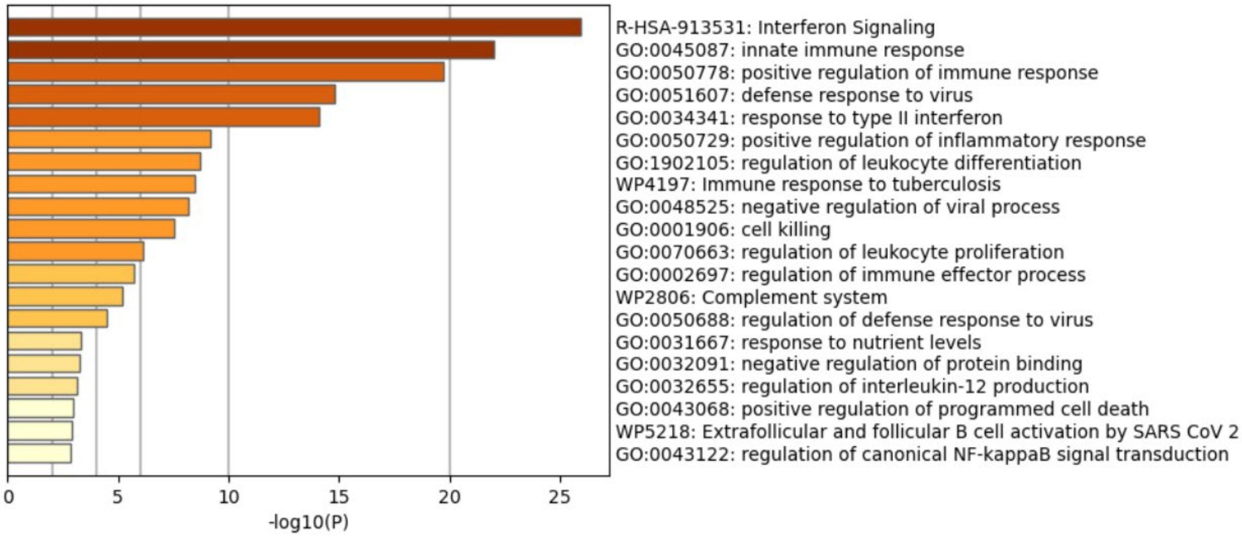

Donor 2 Cluster 6

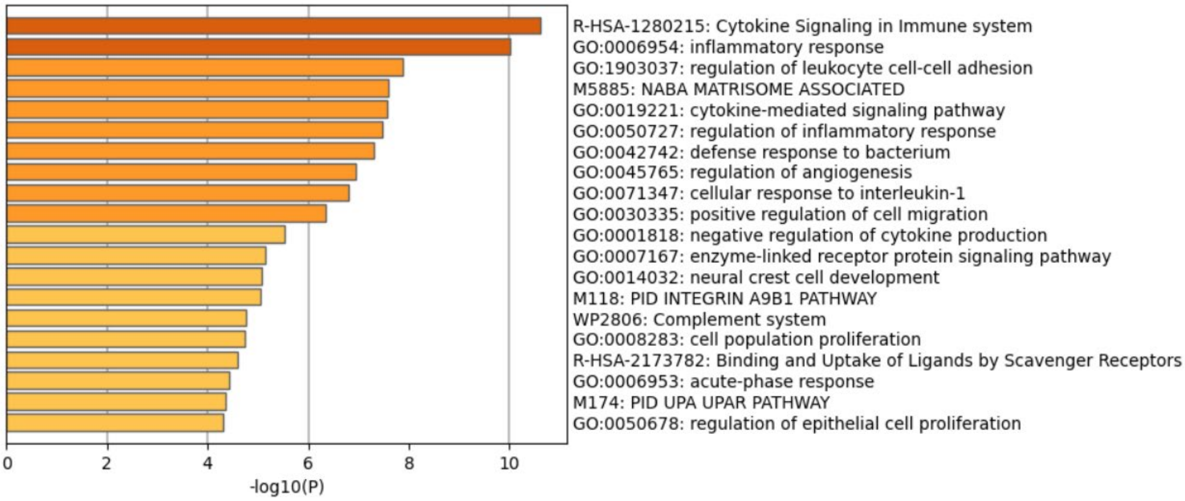

Donor 2 Cluster 7

Only 91 genes were categorized in this cluster, Functional annotation analysis was not conducted.
