# Supplementary material for: Interleukin-27-polarized HIV-resistant M2 macrophages are a novel subtype of macrophages that express distinct antiviral gene profiles in individual cells: implication for the antiviral effect via different mechanisms in the individual cell-dependent manner
Source: Front Immunol. 2025 Mar 10;16:1550699. doi: 10.3389/fimmu.2025.1550699 (PMC11931227; doi:10.3389/fimmu.2025.1550699)
Supplement: Supplementary file 20 [file Image7.pdf]

Supplemental Figure S7

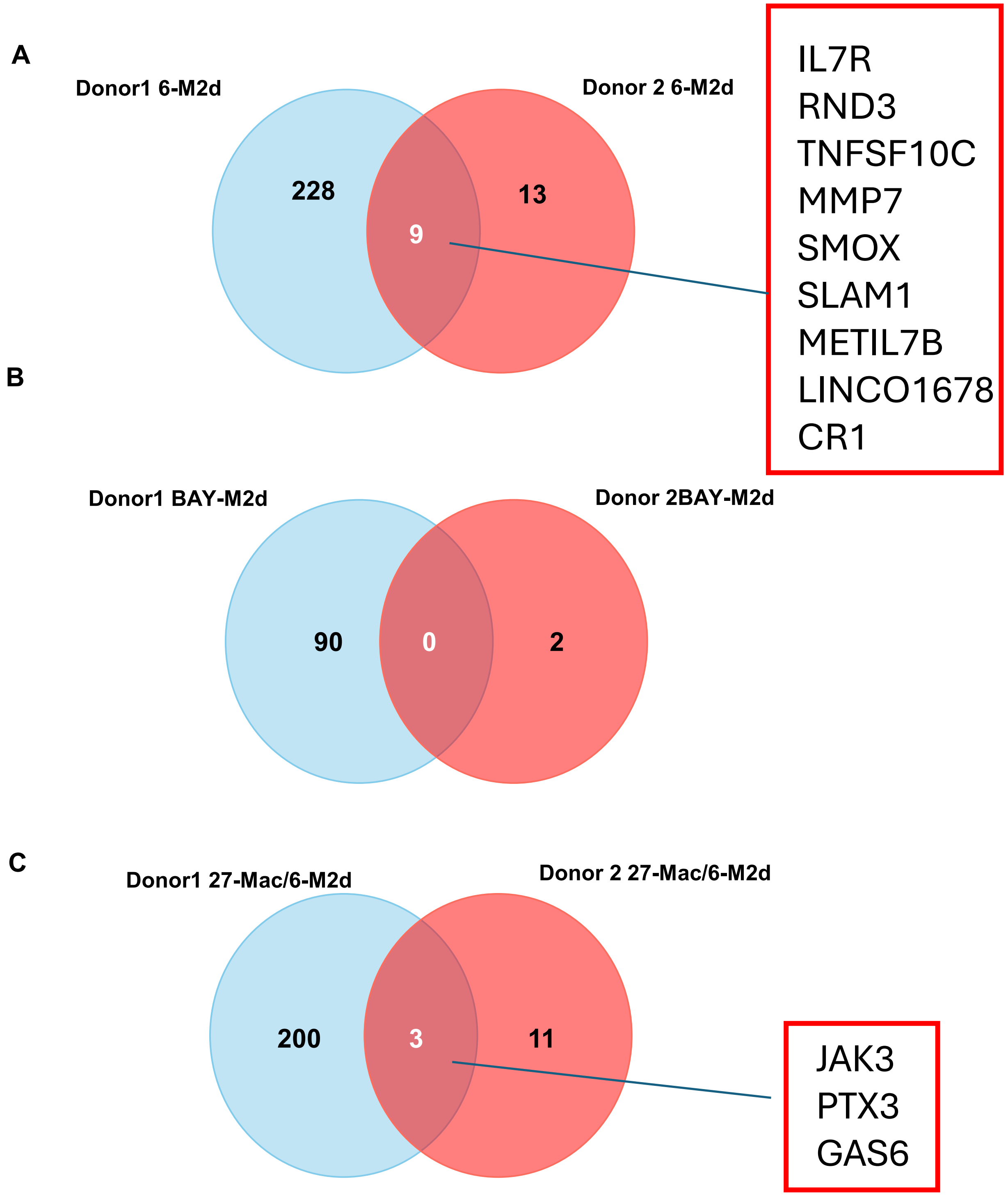

(A, B) Unique DEGs in 6M2d (A), or BAY-M2d (B) were identified using Venn diagram.
(C) The common DEGs to 27Mac and 6M2d were defined using Venn diagram.

Total 203 (130 +73) genes from Donor1 and 14 genes from Donor 2 were subjected.
